# Early-phase drug discovery of β-III-spectrin actin-binding modulators for treatment of spinocerebellar ataxia type 5

**DOI:** 10.1016/j.jbc.2023.102956

**Published:** 2023-01-31

**Authors:** Piyali Guhathakurta, Robyn T. Rebbeck, Sarah A. Denha, Amanda R. Keller, Anna L. Carter, Alexandra E. Atang, Bengt Svensson, David D. Thomas, Thomas S. Hays, Adam W. Avery

**Affiliations:** 1 Department of Biochemistry, Molecular Biology and Biophysics, University of Minnesota, Minneapolis, Minnesota, USA; 2 Department of Chemistry, Oakland University, Rochester, Michigan, USA; 3 Department of Genetics, Cellular Biology, and Development, University of Minnesota, Minneapolis, Minnesota, USA

**Keywords:** spinocerebellar ataxia, SCA5, β-III-spectrin, drug screening, actin binding, time-resolved FRET, fluorescence lifetime, swinholide A, ABD, actin-binding domain, CH, calponin homology, DMSO, dimethyl sulfoxide, F-actin, actin filaments, FLT, fluorescent lifetime, FRET, fluorescence resonance energy transfer, HTS, high-throughput screening, PR, plate reader, SCA5, spinocerebellar ataxia type 5

## Abstract

β-III-Spectrin is a key cytoskeletal protein that localizes to the soma and dendrites of cerebellar Purkinje cells and is required for dendritic arborization and signaling. A spinocerebellar ataxia type 5 L253P mutation in the cytoskeletal protein β-III-spectrin causes high-affinity actin binding. Previously we reported a cell-based fluorescence assay for identification of small-molecule actin-binding modulators of the L253P mutant β-III-spectrin. Here we describe a complementary, *in vitro*, fluorescence resonance energy transfer (FRET) assay that uses purified L253P β-III-spectrin actin-binding domain (ABD) and F-actin. To validate the assay for high-throughput compatibility, we first confirmed that our 50% FRET signal was responsive to swinholide A, an actin-severing compound, and that this yielded excellent assay quality with a Z′ value > 0.77. Second, we screened a 2684-compound library of US Food and Drug Administration–approved drugs. Importantly, the screening identified numerous compounds that decreased FRET between fluorescently labeled L253P ABD and F-actin. The activity and target of multiple Hit compounds were confirmed in orthologous cosedimentation actin-binding assays. Through future medicinal chemistry, the Hit compounds can potentially be developed into a spinocerebellar ataxia type 5–specific therapeutic. Furthermore, our validated FRET-based *in vitro* high-throughput screening platform is poised for screening large compound libraries for β-III-spectrin ABD modulators.

β-III-Spectrin is a key actin-cross-linking protein that localizes to the dendrites and soma of cerebellar Purkinje cells (1). Mouse models showed that β-III-spectrin is required for Purkinje cell dendritic arborization and proper postsynaptic localization of multiple membrane proteins (2, 3). Autosomal dominant mutations in *SPTBN2* gene encoding β-III-spectrin cause the neurodegenerative disease, spinocerebellar ataxia type 5 (SCA5) (4). SCA5 causes degeneration of Purkinje cells and an associated, progressive limb and gait ataxia. Currently there is no cure or therapy for SCA5.

SCA5-associated mutations localize to the β-III-spectrin N-terminal actin-binding domain (ABD) and spectrin-repeat domains. We previously showed that the ABD-localized L253P mutation causes high-affinity actin binding (5). L253P is positioned at the interface of the two calponin homology domains (CH1 and CH2) comprising the ABD. Our biochemical and biophysical studies showed that L253P causes high-affinity actin binding by opening the CH1–CH2 interface, allowing CH1 to directly bind actin (6). This high-affinity actin binding results in mislocalization of the mutant L253P β-III-spectrin in dendrites (7, 8). This mislocalization would be expected to interfere with β-III-spectrin’s role as a scaffold for postsynaptic proteins, including glutamate transporters and receptors, as established by β-III-spectrin mouse studies (9, 10). Numerous additional SCA5 mutations localize to the CH1–CH2 interface, suggesting that high-affinity actin binding may be a shared molecular consequence of the ABD-localized SCA5 mutations (11). Thus, it is likely that a small-molecule compound that reduces binding of β-III-spectrin L253P mutant to actin will be effective as a SCA5 therapeutic for other ABD mutants. Such a small molecule would restore proper dendritic localization of β-III-spectrin and its role as a scaffold for the postsynaptic membrane proteins.

We previously reported the development of a cell-based high-throughput screening (HTS) platform to identify small-molecule actin-binding modulators of the L253P ABD (12). In this assay, HEK293 cells are transiently transfected with GFP-ABD (donor) and an actin-binding peptide, Lifeact, that is N-terminally labeled with mCherry (acceptor). Binding of GFP-ABD and Lifeact-mCherry on neighboring actin protomers resulted in ∼12% FRET efficiency. Specificity of the FRET signal was demonstrated by the actin-severing compound swinholide A, which greatly reduced the FRET signal. Screening of a 1280-compound library identified Hits that reduced Lifeact binding to actin, and showed that the assay is sensitive to compounds that cause cell lysis (12).

Here we report the development of a complementary and more sensitive FRET-based HTS platform that uses purified L253P ABD and F-actin. Key features include that the assay 1) has greatly increased FRET efficiency, 2) consists of only two components (fluorescently labeled ABD and actin), and 3) is insensitive to compound cytotoxicity and poor cell permeability. Screening of a library of US Food and Drug Administration (FDA)-approved compounds and further evaluation with cosedimentation assays established the validity of the current assay for detection of small molecules that reduce binding of the L253P ABD to actin.

## Results

### Biosensor validation for high-throughput compatibility by swinholide A

**Figure 1:**
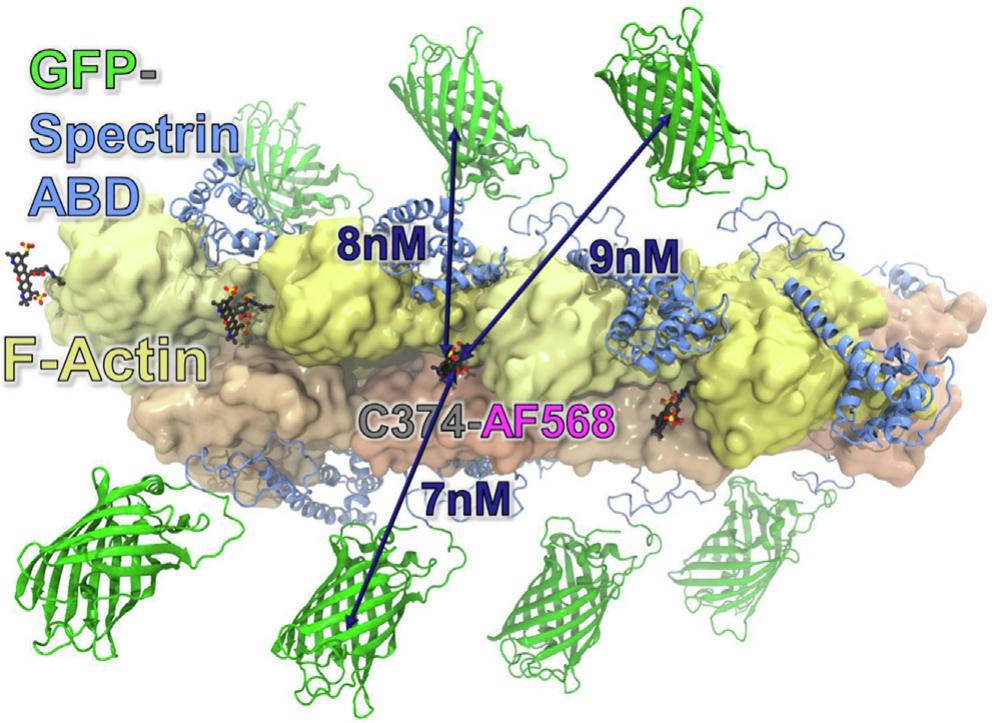
**Model of *in vitro* actin-binding domain biosensor.** Model of mNeonGreen(GFP)-ABD-L253P bound to F-actin labeled with AF568 at C374. Position of β-III-spectrin ABD L253P on actin was based on the cryo-EM structures 6ANU (6), and modeled using DS Visualizer (Dassault Systemes). Molecular figure was generated using VMD (41).

**Figure 2:**
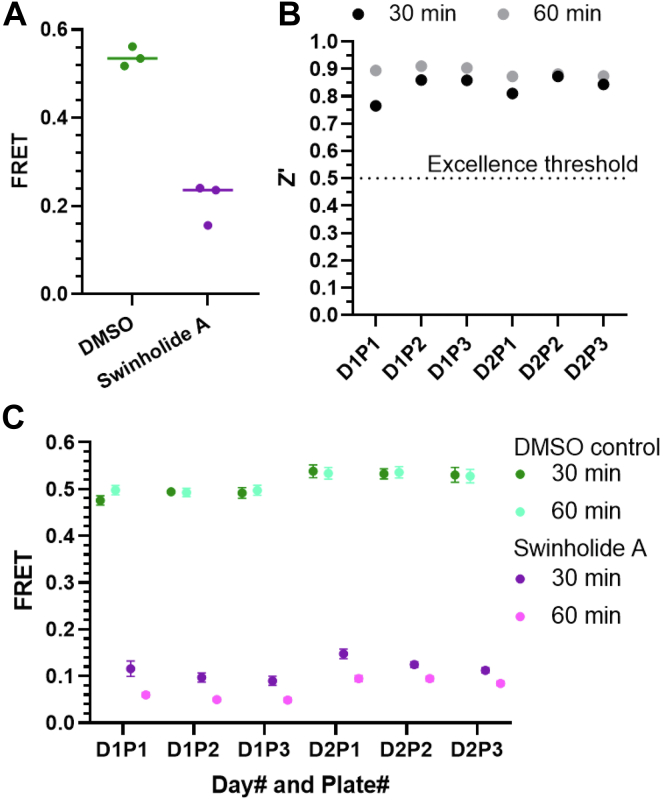
**Swinholide A is a reproducible, positive control tool compound for FRET assays in 1536-well plates.***A*, FRET between mNG-ABD-L253P and AF568-actin is reduced by swinholide A. For each plate, each condition was loaded over 27 wells on a 1536-well plate, n = 3 plates. *B* and *C*, plot of Z′-factor (*B*) and FRET (*C*) value per 1536-well plate over 2 days for 5 μM swinholide A loaded over 32 wells *versus* dimethyl sulfoxide (DMSO) control loaded over 224 wells. Fluorescence lifetimes were acquired at the 30 and 60 min time points after addition of swinholide A or DMSO control. Data shown as mean ± SD (*C*).

To complement our live cell ABD biosensor, we developed an *in vitro* FRET-based biosensor using purified ABD and F-actin. A FRET donor construct consisting of the green fluorescent protein, mNeonGreen (mNG), fused to the N terminus of the mutant ABD (mNG-ABD-L253P), was incubated with the phalloidin-stabilized filamentous actin (F-actin). Actin was labeled with an acceptor dye Alexa Flour 568 (AF568) at residue C374. With mNG-ABD-L253P bound to F-actin, we predicted that each of the mNG fluorescent proteins (donor) would be within 7 to 9 nm distance of AF568 (acceptor) on actin-C374, on the same actin monomer and the neighboring actin monomers (Fig. 1). mNG-ABD-L253P was excited with a 473-nm laser, and FRET was measured as a reduction in the fluorescence lifetime (FLT) of the donor (τ_D_), in the presence of the acceptor, AF568-actin. With multiple FRET acceptors within the range of the FRET donor, this aligned with the high level (54%) of FRET observed following 20 min of incubation of 0.5 μM mNG-ABD-L253P and 1 μM AF568-actin (Fig. 2*A*).

To test the compatibility of mNG-ABD-L253P and AF568-actin FRET assay for the primary compound screening, we tested the response of our biosensor to the F-actin severing compound, swinholide A. This compound was previously established as a tool compound for our live cell FRET assay (12). As shown in Figure 2*A*, 20-min incubation with swinholide A reduced the FRET signal by 60.0 ± 10.2%. Thus, swinholide A was used, here, as a tool compound in the current assay for further characterization.

To gauge HTS assay robustness, we used swinholide A and the FRET biosensor in 1536-well plates to measure the Z' value, which factors the signal window and data variation between control and tool compound effect. Classically, a value of 0.5 ≤ Z'< 1 indicates an excellent assay that is ready for large-scale HTS (13). For evaluating reproducibility, we acquired the FLT values, at 30 and 60 min post loading, on three plates per day for 2 days. As shown in Figure 2*B*, the Z' per plate ranged from 0.77 to 0.91, easily exceeding the 0.5 Z′-factor excellence threshold. Reading the FLT values at 60 min instead of 30 min post plate loading marginally increased the Z′ value by 6.7% (*p* = 0.022). This is reflected by the slightly larger effect of swinholide A at the longer time point (Fig. 2*B*). Importantly, FRET values were highly reproducible when measured in repeat tests performed on the same day, or on different days, using different preparations of AF568-actin (Fig. 2*C*). Overall, these results indicate the compatibility of the assay for HTS.

### HTS performance

To test the performance of the FRET assay in HTS, we screened the 2684-compound Selleck FDA and clinical drug library for modulators that alter actin binding of the β-III-spectrin ABD L253P mutant. This library is desirable for pilot screening, as the library compounds all have a rich research history and may directly lead into further studies into therapeutic potential for SCA5. The compounds, together with dimethyl sulfoxide (DMSO) controls, were dispensed in 15 nl volumes into individual wells of three 1536-well microplates and stored at −20 °C until use. Following plate thaw, 5 μl of mNG-ABD L253P alone (donor only), or mNG-ABD L253P and AF568-actin (donor + acceptor) assay mix were loaded into each well *via* a multidrop liquid dispenser. The final compound concentration equaled 30 μM. Plate loading times were staggered to allow for 6-min FLT acquisition time between plates. A time course of compound effects (at 30 and 60 min post load) on FLT was acquired using a high-speed, high-precision fluorescence lifetime plate reader. This technology has been advanced to high-density 1536-well plates in recent years for successful HTS using a range of protein biosensors including β-III-spectrin ABD in mammalian cells (12), sarcoplasmic reticulum Ca-ATPase (14, 15, 16, 17), ryanodine receptor (18, 19), actin (20), cardiac myosin-binding protein C (21), tumor necrosis factor receptor 1 (22, 23), and tau (24).

**Figure 3:**
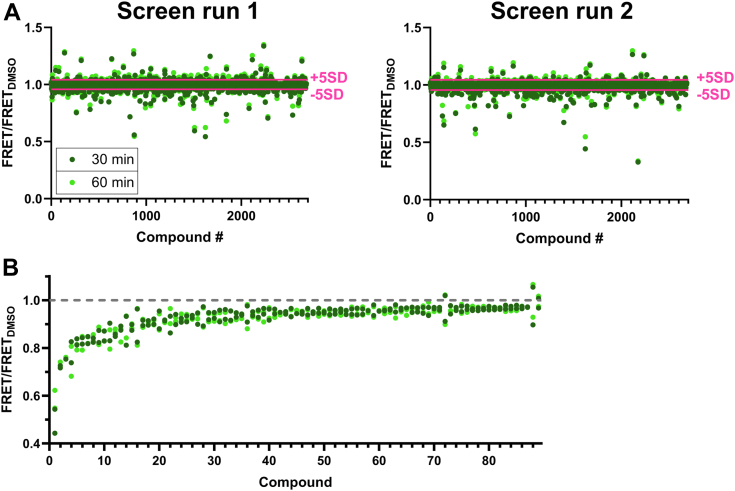
**High-throughput screening performance validation of *in vitro* actin-binding domain biosensor FRET assay using the Selleck Clinical and FDA-approved drug library in 1536-well plates.** Fluorescence lifetime data were acquired at two time points following FRET assay loading into 1536-well plates that were preloaded with 2684-compound library (30 μM final) or dimethyl sulfoxide (DMSO) control. *A,* FRET response to Selleck compounds, with interfering compounds removed, for run 1 (*left panel*) and run 2 (*right panel*). FRET response beyond the 5 standard deviation (SD) threshold (*pink line*) demonstrates that most Hit compounds have little time-dependent effect. *B,* relative FRET effect of Selleck Hits that were identified (with 5 SD threshold) as decreasing FRET in both screen runs, with an additional 17 compounds added due to potent effect (>7 SD) in one screen. Data shown as relative to DMSO control (*gray dotted line*). Chemical names and structures shown in Figs. S2–S11.

Interfering fluorescent compounds were identified as compounds that altered the fluorescence spectrum by >3 SD, as previously described (14, 15, 18, 19, 25). The effects of the compounds on the FRET biosensor are shown in Figure 3. For most compounds, there was little variation in the magnitude of the FRET effect at the 30 or 60 min incubation time (Fig. 3*A*). This is also reflected by the similar number of compounds that altered FRET at different thresholds (3, 5, or 7 standard deviations of the DMSO control mean), at either time point (Table 1).

**Table 1:** Number (#) of Hits and Hit reproducibility for 3, 5, and 7 standard deviation (SD) thresholds

Standard deviation	Hit evaluation method	SELLECK run 1	SELLECK run 2
3 SD	# of Hits 30 min (hit rate%)	492 (18.6%)	498 (18.8%)
	# of Hits 60 min (hit rate%)	523 (19.8%)	501 (18.9%)
	% of Repeated Hits in 2 runs—30 min	45.3%	44.8%
	% of Repeated Hits in 2 runs—60 min	49.5%	51.7%
5 SD	# of Hits 30 min (hit rate%)	247 (10.3%)	248 (9.4%)
	# of Hits 60 min (hit rate%)	288 (10.9%)	250 (9.5%)
	% of Repeated Hits in 2 runs—30 min	38.5%	38.0%
	% of Repeated Hits in 2 runs—60 min	38.2%	44.0%
7 SD	# of Hits 30 min (hit rate%)	164 (6.3%)	155 (5.8%)
	# of Hits 60 min (hit rate%)	155 (5.9%)	145 (5.5%)
	% of Repeated Hits in 2 runs—30 min	29.3%	31.0%
	% of Repeated Hits in 2 runs—60 min	36.8%	39.3%

To cast a wide net for possible modulators, we focused on compounds that reproducibly altered FRET by the 5 SD threshold. Notably, the resulting 135 compounds is a higher number of compounds than the optimal rate previously described (<3%) (26). However, this is likely the result of having a high FRET signal window (∼54%) relative to previous biosensors (<20%) (12, 18, 19). An additional contributing factor is that we did not remove compounds that alter donor-only FLT, in order to include potential compounds that impact donor-only FLT in the process of binding and structurally modifying the ABD.

In addition to the reproducible compounds, we focused on 17 compounds that potently (>7 SD) altered FRET in one of the two screens. Thus, prioritized compounds were selected from a list of 152 compounds, termed “Hits,” from the screens. Considering the average of the compound effects, this resulted in 89 FRET decreasers and 63 FRET increasers (Figs. 3*B* and S1–S16).

An additional factor that may contribute to the higher Hit percentage is that this library contains different salt forms for several compounds and several groups of similar chemical types. Indeed, we identified several salt forms and analogues of chemical types or drug classes. Because our ultimate interest is to identify compounds that reduce the affinity of mutant β-III-spectrin for actin, we further characterized the Hits that reduced FRET.

### FRET dose–response assay

**Figure 4:**
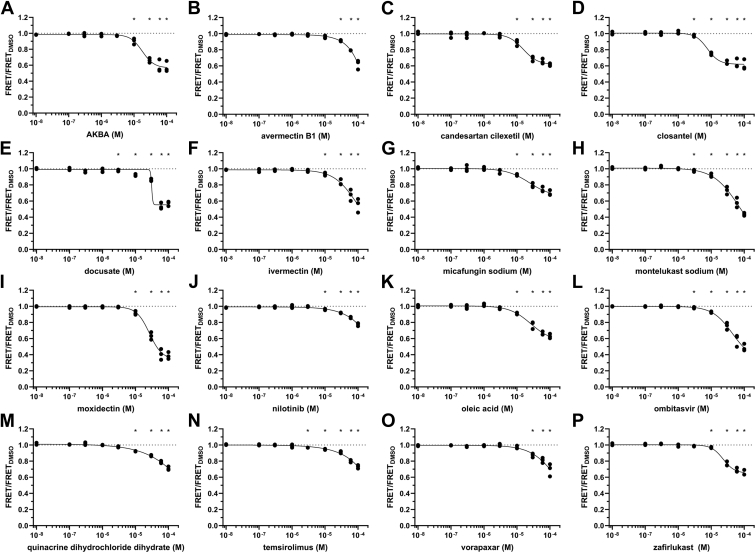
**FRET dose–response of Hit compounds that decrease FRET by greater than 20%.***A*–*P*, Dose–response of Hit compounds AKBA (*A*), avermectin B1 (*B*), candesartan (*C*), closantel (*D*), docusate (*E*), ivermectin (*F*), micafungin (*G*), montelukast (*H*), moxidectin (*I*), nilotinib (*J*), oleic acid (*K*), ombitasvir (*L*), quinacrine (*M*), temsirolimus (*N*), vorapaxar (*O*), and zafirlukast (*P*) were tested on the *in vitro* actin-binding domain biosensor FRET. The chemical structures of these Hit compounds are shown in Figs. S3–S16. ∗*p*< 0.05 relative to dimethyl sulfoxide (DMSO) control, using Student’s unpaired *t* test. Data shown as individual data points, n = 3.

Thirty-eight Hits that showed the most potent response for their salt form or drug class on FRET were selected for purchase and further validation by first acquiring the FRET response to a range of Hit compound concentrations (0.01–100 μM) under the same assay conditions as used in the primary screen. As shown in Figure 4, 16 of the repurchased Hits reduced FRET by >20%, notably with similar effects as observed in the primary screens. Of the remaining 22 compounds, 19 compounds significantly reduced FRET (Fig. S17). Encouragingly, 92% of Hits were verified upon repurchase.

### Confirmation of Hit compound activity in orthologous binding assay

**Figure 5:**
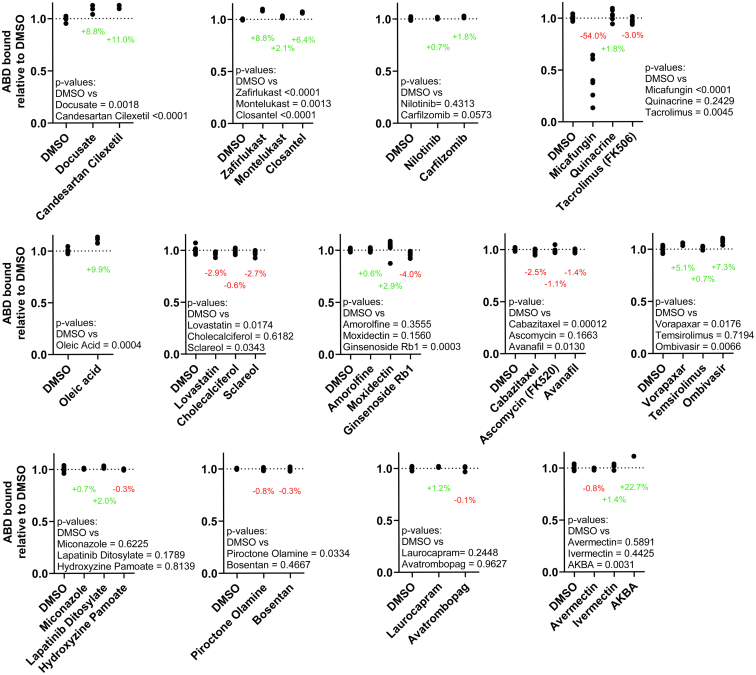
**Confirmation of Hit compounds in actin cosedimentation assays.** Cosedimentation of L253P actin-binding domain (ABD) and actin shows that most Hit compounds (100 μM) decrease or increase cosedimentation of the L253P ABD (2 μM) with F-actin (1.2 μM). Data are shown as relative to dimethyl sulfoxide (DMSO), n= 3 to 12.

To confirm the activity of Hits to reduce binding of the L253P ABD to F-actin, cosedimentation assays were performed using purified ABD and F-actin (Fig. 5). ABD, 2 μM, was incubated with 1.2 μM F-actin in the presence of 100 μM compound or DMSO. Most of the 33 compounds that reduced binding in the FRET assay either decreased or increased cosedimentation of the mutant ABD with F-actin.

**Figure 6:**
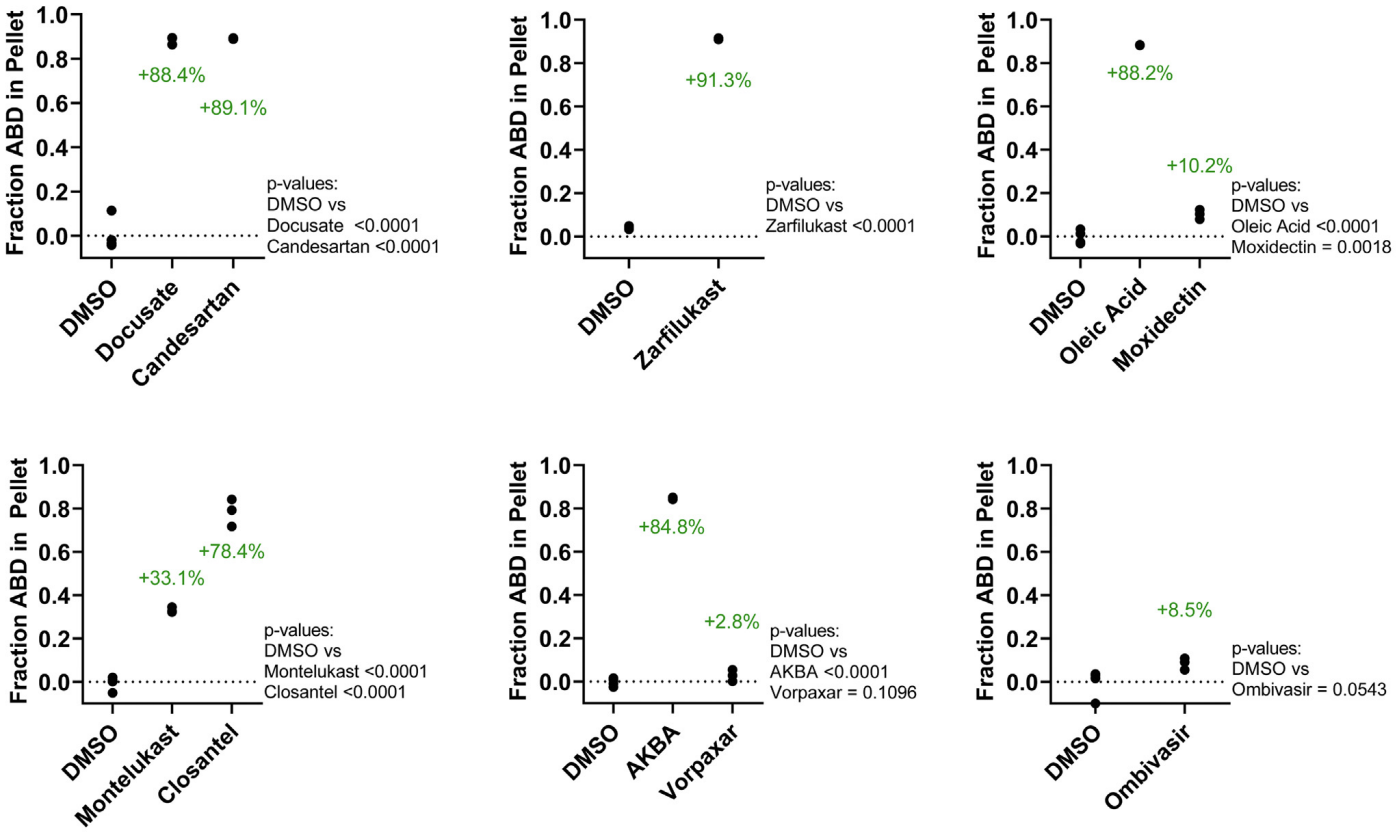
**Aggregation assay of Hit compounds.** For Hit compounds that caused the L253P actin-binding domain (ABD) to enter pellet in actin cosedimentation assays, cosedimentation reactions were repeated in the absence of actin. All tested compounds (100 μM) caused the L253P ABD to enter pellet in the absence of actin. Data are shown as relative to dimethyl sulfoxide (DMSO), n= 3 to 6.

Eleven compounds caused a significant (2–22%) increase in cosedimentation of the ABD with actin. This increase in ABD cosedimentation reflects either an effect of the compounds to increase binding of the ABD to F-actin (counter the FRET results) or compound-induced aggregation of the mutant ABD. To examine the aggregation effects of these compounds, we tested whether the compounds caused ABD sedimentation in the absence of actin (Fig. 6). All of the compounds caused the ABD to enter the pellet in the absence of actin, confirming that the Hit compounds were causing ABD aggregation. Consistently, many of the compounds that we experimentally determined to cause ABD aggregation are known or predicted aggregators in the Aggregator Database (27) (Table S1).

**Figure 7:**
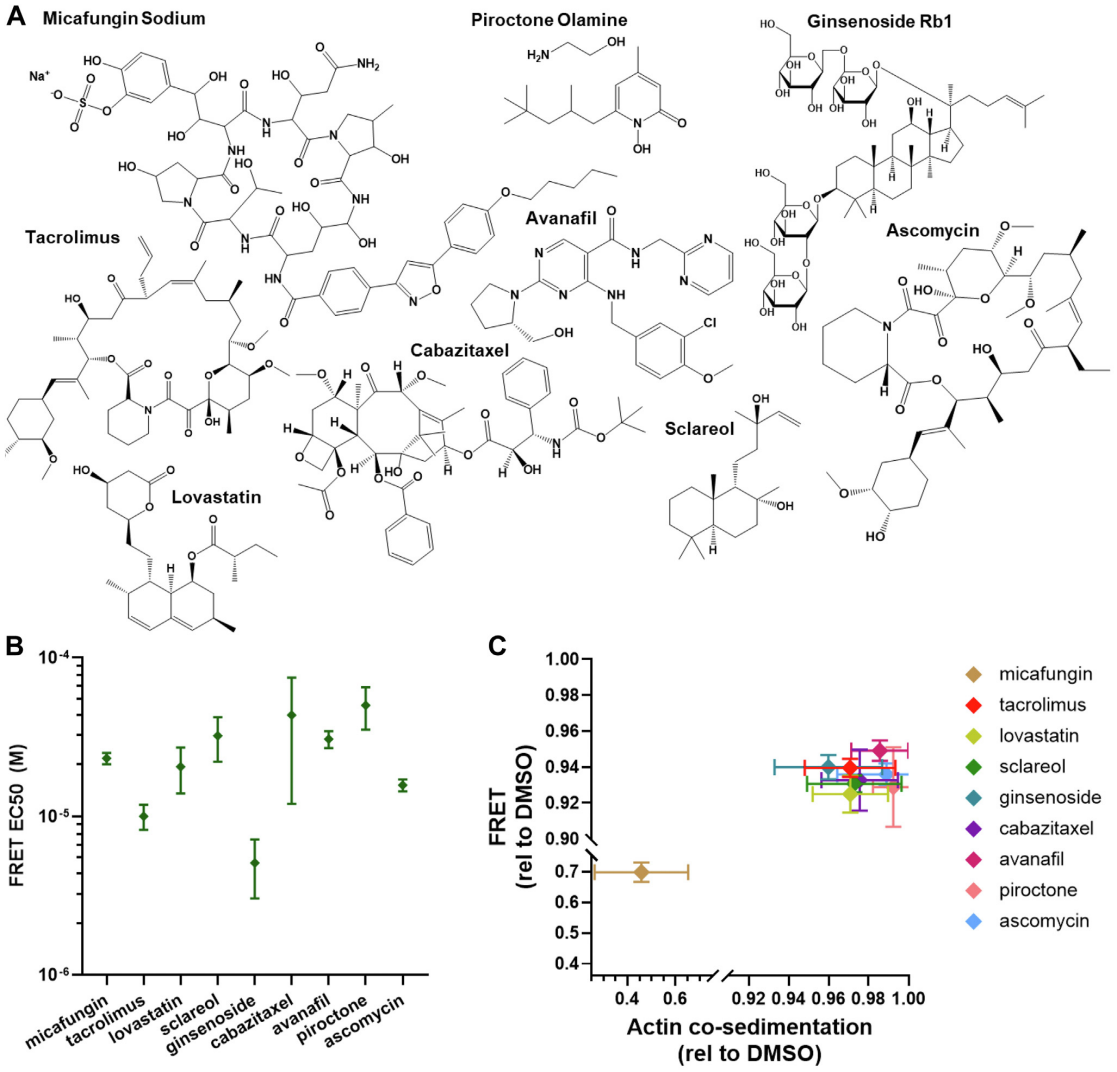
**Summary of compounds that reduce actin-binding domain–actin binding.***A*, compound structures. *B*, FRET EC_50_ from Hill fits of data shown in Figure 4 and Fig. S17. Data shown as mean ± SD, n = 3. *C*, comparison of 100 μM compound on actin cosedimentation *versus* FRET, data shown in Figures 4 and 5, and Fig. S17. Data shown as mean ± SD.

Nine compounds reduced actin binding in cosedimentation assays significantly, by ∼1 to ∼50%. Of these compounds, Micafungin had the largest effect, decreasing ABD cosedimentation by ∼50%. Other compounds that caused a significant reduction in ABD cosedimentation included Tacrolimus, Lovastatin, Sclareol, Ginsenoside Rb1, Cabazitaxel, Avanafil, and Piroctone (Fig. 5). Ascomycin also caused average ABD binding to be reduced, although the effect fell short of statistical significance. For these compounds that reduced ABD binding in the cosedimentation assays, similar effect sizes were observed in the FRET binding assays (Figs. 4, 7 and S17). Of the nine compounds, Ginsenoside Rb1 had the lowest EC_50_, ∼3 μM (Figs. 7 and S17). None of the Hit compounds that reduced ABD cosedimentation are known or similar to known aggregators in the Aggregator Database (Table S1). These nine Hit compounds are thus of greatest interest as potential modulators of L253P mutant β-III-spectrin actin-binding activity.

## Discussion

We have developed a high-precision, FRET-based HTS assay that monitors binding of recombinant mNeonGreen-tagged L253P β-III-spectrin ABD to actin filaments covalently linked to a fluorescent dye Alex Fluor 568. In this assay, binding of the L253P ABD to actin filaments results in ∼50% FRET efficiency. This FRET signal is abolished by the F-actin severing compound, swinholide A, in agreement with our prior cryo-EM model showing that ABD actin binding involves contact with two adjacent actin subunits within the filament (6). The large FRET signal makes the assay amenable to HTS, as indicated by the high Z′ values achieved when the assay is performed in 1536-well microplates.

We validated the assay for HTS by screening Selleck’s 2684-compound FDA and clinical library. Duplicate screens led to the identification of nine compounds with confirmed activity to decrease L253P ABD and actin binding. This highlights the effectiveness of this assay at identifying compounds with desirable activity. In addition, these Hit compounds support that the ABD-actin interaction is a druggable target. The successful identification of Hit compounds in this small compound library screen warrants further screening of larger compound libraries to increase the number and structural diversity of the Hit compounds. Notably, numerous spectrin-related proteins, including α-actinins (28) and filamins (29), contain disease-associated mutations in their ABDs. The HTS platform we have validated here for β-III-spectrin should be easily adaptable for drug discovery targeting these related disease proteins.

Of the nine Hit compounds that reduced L253P ABD and actin binding (Figs. 5 and 7), micafungin stands out as having the largest effect size, reducing actin binding by ∼50%. Micafungin is a semisynthetic compound approved by the FDA as an antifungal agent that works by inhibiting synthesis of the fungal cell wall component, 1,3-β-D-glucan (30). Micafungin is intravenously injected to treat systemic fungal infections and is reported to be tolerated at high dose with little adverse side effect (31). Micafungin’s large size (1292.26 Da) probably limits its ability to cross the blood–brain barrier. However, Micafungin can be detected at low level in the central nervous system (32).

Ginsenoside Rb1 stood out as the Hit with the lowest EC_50_, ∼3 μM, and reduced actin binding by 4 to 6% in actin cosedimentation and FRET assays. Ginsenosides are bioactive components of the ginseng plant. Intriguingly, numerous studies in mammalian cell systems suggest that Ginsenoside Rb1 has neuroprotective properties (33). For example, Ginsenoside Rb1 was reported to ameliorate motor deficits in a mouse model of Parkinson’s disease, potentially by reducing glutamate-mediated neurotoxicity by upregulated expression of the GLT-1 glutamate transporter (34). Purkinje cell loss in SCA5 pathogenesis has also been linked to glutamate-mediated neurotoxicity (35, 36). It is appealing to hypothesize that Ginsenoside Rb1 could work as a SCA5 therapeutic by ameliorating aberrant β-III-spectrin actin binding, thereby restoring β-III-spectrin localization and role as a scaffold for postsynaptic proteins. Downstream, this could restore clearance of glutamate. Independently, Ginsenoside Rb1 could enhance glutamate clearance by upregulation of glutamate transporters. Thus, Ginsenoside Rb1 would work as a SCA5 therapeutic by two independent mechanisms.

Currently, we are in the early phase of SCA5 therapeutic discovery. While the therapeutic potential of current Hit compounds is supported by our *in vitro* assays, we emphasize that none of Hit compounds have been evaluated for efficacy or safety in treating neurodegeneration or ataxia associated with SCA5, in any system. For our current Hit compounds, medicinal chemistry may be necessary to increase compound potency (reduced EC_50_ and/or increased effect size). Moreover, the activity of the compounds should be tested in cell models that have revealed mislocalization of the L253P mutant β-III-spectrin (7, 8). To assess Hit compound therapeutic potential, it is now essential that a SCA5 L253P mouse model be generated. With this mouse model, it would then be feasible to test these compounds on arborization of cultured neurons or whole mouse ataxia phenotype. Furthermore, screening of additional, larger compound libraries would increase the number and structural diversity of Hits, ensuring successful development of a SCA5 therapeutic.

## Experimental procedures

### Expression and purification of mNeonGreen and mNeonGreen-ABD proteins

The mNeonGreen coding sequence was synthesized at Integrated DNA Technologies and matches GenBank sequence KC295282.1. mNeonGreen was digested with KpnI and EcoRI and subcloned into pcDNA3.1-GFP-ABD L253P (37), after digestion with KpnI and EcoRI to remove GFP. mNeonGreen (mNG) and mNeonGreen-ABD L253P (mNG-ABD L253P) coding sequences were PCR amplified with the forward primer AAACACCTGCAAAAAGGTATGGTGAGCAAGGGCGAGGAGG, and the reverse primer AAATCTAGACTACTTGTACAGCTCGTCCATGCCC for mNG, or the reverse primer AAATCTAGACTACTTCATCTTGGAGAAGTAATGGTAGTAAG for mNG-ABD L253P. Following digest with Aarl and Xbal, PCR products were ligated into BsaI-digested pE-SUMOpro (LifeSensors). Sequence-verified constructs were transformed into Rosetta 2 (DE3) *Escherichia coli* (Novagen). mNG and mNG-ABD L253P protein expression and purification steps, including removal of SUMO tag, were performed as described (37). A final step of buffer exchange was performed for mNG by dialysis in buffer containing 10 mM Tris, pH 7.5, 150 mM NaCl, 2 mM MgCl_2_, and 1 mM DTT using Slide-A-Lyzer, 10,000 MWCO cassettes (Thermo Scientific). For mNG-ABD L253P a final gel filtration step was performed using a S-100 Sephacryl column equilibrated with buffer containing 10 mM Tris, pH 7.5, 150 mM NaCl, 2 mM MgCl_2_, and 1 mM DTT. Fractions containing mNG-ABD L253P were concentrated using a Centrifugal Filter Unit, 10,000 MWCO (Millipore). mNG and mNG-ABD L253P protein concentrations were determined through Bradford assay. Purified proteins were supplemented with sucrose (150 mM final), snap frozen in liquid nitrogen, and stored at −80 °C until use.

### Actin preparation and labeling

Actin was prepared from rabbit skeletal muscle by extracting acetone powder in cold water, as described (38). Alexa Fluor 568 C5 maleimide (Invitrogen), 130 μM, freshly dissolved in dimethylformamide, was added to 65 μM F-actin, and the sample was incubated for 30 min at 25 °C and then 18 h at 4 °C. Labeling was terminated by adding 10 mM DTT, and actin was ultracentrifuged for 30 min at 350,000*g*. The F-actin pellet was suspended in G-Mg buffer (5 mM Tris, 0.5 mM ATP, 0.2 mM MgCl_2_, pH 7.5) followed by clarification at 300,000*g* for 10 min. Actin was again polymerized for 45 min at 25 °C in the presence of 3 mM MgCl_2_ and ultracentrifuged at 350,000*g* for 30 min. The F-actin pellet was suspended in F-Mg buffer (3 mM MgCl_2_, 10 mM Tris, pH 7.5) containing 0.2 mM ATP. The labeled F-actin was immediately stabilized against depolymerization and denaturation by adding equimolar phalloidin.

### Fluorescence data acquisition

Fluorescence lifetime measurements were carried out by a high-precision FLTPR (provided by Photonic Pharma LLC) (15, 39). Donor sample (mNG-β-III-spectrin ABD L253P) was excited with a 473-nm microchip laser (Bright Solutions), and emission was filtered with 488-nm long pass and 517/20-nm band pass filters (Semrock). This instrument enables high-throughput fluorescence lifetime detection at high precision by utilizing a unique direct waveform recording technology (15). The performance of this FLTPR has been previously demonstrated with FRET-based HTS that targets several muscle and nonmuscle proteins (12, 15, 18). In the present study, modifications were made in the instrument to permit two-channel detection, for the purpose of flagging false Hits due to interference from fluorescent compounds.

### Screen with SELLECK library

The 2684 SELLECK compounds were received in 96-well plates and reformatted into 1536-well flat, black-bottom polypropylene plates (Greiner Bio-One). In total, 50 nl of each compound solution was dispensed in DMSO using an automated Echo 550 acoustic liquid dispenser (Labcyte). Compounds were formatted into the assay plates, at a final concentration of 30 μM, with the first two and last two columns loaded with DMSO only (compound-free controls). These assay plates were then heat-sealed using a PlateLoc Thermal Microplate Sealer (Agilent Technologies) and stored at −20 °C. Before screening, compound plates were equilibrated to room temperature (25 °C). In total, 0.5 μM mNG-ABD1 without or with 1 μM Alexa-568-labeled actin was dispensed by a Multidrop Combi Reagent Dispenser (Thermo Fisher Scientific) into the 1536-well assay plates containing the compounds. Plates were incubated at room temperature for 60 min before recording the data with the FLTPR. A control measurement with 0.5 μM mNG (fluorescent protein only) was performed to eliminate the compounds that affected the environment of the fluorescence protein.

### HTS data analysis

Waveforms for each well in HTS were convolved with the instrument response function and were fitted by a one-exponential decay function using least-squares minimization (40). The FRET efficiency (*E*) was determined as the fractional decrease of donor fluorescence lifetime (τ_D_), due to the presence of acceptor fluorophore (τ_DA_):

(1) $$E=1-\frac{\tau_{DA}}{\tau_{D}}$$

Assay quality was determined based on FRET assay samples in wells preloaded with control (DMSO) and tested tool compound, as indexed by the Z′ factor:

(2) $$Z^{\prime }=1-3\frac{\sigma_{DMSO}+\sigma_{Tool}}{\left|\mu_{DMSO}-\mu_{Tool}\right|}$$

where σ_DMSO_ and σ_Tool_ are the SDs of the DMSO τ_DA_ and tool compound τ_DA_, respectively; μ_DMSO_ and μ_Tool_ are the means of the DMSO τ_DA_ and tool compound τ_DA_, respectively. A compound was considered a Hit if it changed τ_DA_ by > 5 SD relative to that of control τ_DA_ that were exposed to 0.3% DMSO.

### Compound’s concentration–response assay

The Hit compounds were purchased and dissolved in DMSO to make a 10 mM stock solution, which was serially diluted in 96-well mother plates. Hits were screened at nine concentrations (0.01–100 μM), with DMSO controls also loaded. Compounds (1 μl) were transferred from the mother plates into 1536-well plates using a Mosquito HV liquid handler (TTP Labtech Ltd). The same procedure of dispensing as for the pilot screening was applied in the TR-FRET concentration–response assays. The FRET efficiency E was determined as the fractional decrease in donor fluorescence lifetime as described above. Concentration dependence of the FRET (relative to DMSO control) change was fitted using the Hill equation.

### Cosedimentation

F-actin cosedimentation assays were performed as described (37) with few modifications. After measuring the concentrations of polymerized F-actin and clarified ABD proteins *via* Bradford assay, the binding reactions were set up by combining 2 μM ABD protein, 1.2 μM F-actin, and 100 μM compound or DMSO, to a 60 μl total reaction volume in F-buffer (10 mM Tris, pH 7.5, 150 mM NaCl, 0.5 mM ATP, 2 mM MgCl_2_, and 1 mM DTT). The binding reactions were incubated at 21 °C for 30 min, then centrifuged at 100,000*g* at 25 °C to pellet F-actin. The supernatants containing the unbound ABD were collected and mixed with 4× Laemmli sample buffer. The unbound ABD samples were separated by SDS-PAGE and stained with Coomassie blue R-250 stain for 2 h. Protein band intensities were measured using Image Studio Lite version 5.2 software after imaging the gels using the 680 nm channel on Azure Sapphire scanner. Fraction ABD bound was measured after converting fluorescence intensity measurements to concentrations using a standard curve generated from a Coomassie blue–stained gel containing varying amounts ABD protein in the absence of actin or compound. The data were plotted in Prism 9 (GraphPad) relative to DMSO control average. The aggregation assays were performed as the cosedimentation assays but without F-actin.

### Analysis and presentation of data

Data are presented as mean ± SD. For statistical difference determination, unpaired Student’s *t* test was performed. Statistical analyses were performed with GraphPad Prism and Origin. Significance was accepted at *p* < 0.05. EC_50_ values were derived from fits to Hill equations.

## Data availability

All data are contained within the article.

## Supporting information

This article contains supporting information.

## Conflict of interest

D. D. T. holds equity in, and serves as an executive officer for, Photonic Pharma LLC. These relationships have been reviewed and managed by the University of Minnesota. Photonic Pharma had no role in this study, except to provide some instrumentation, as stated in Experimental Procedures.

## References

[1] Ohara O., Ohara R., Yamakawa H., Nakajima D., Nakayama M. (1998). Characterization of a new beta-spectrin gene which is predominantly expressed in brain. Brain Res. Mol. Brain Res..

[2] Stankewich M.C., Gwynn B., Ardito T., Ji L., Kim J., Robledo R.F. (2010). Targeted deletion of betaIII spectrin impairs synaptogenesis and generates ataxic and seizure phenotypes. Proc. Natl. Acad. Sci. U. S. A..

[3] Clarkson Y.L., Gillespie T., Perkins E.M., Lyndon A.R., Jackson M. (2010). Beta-III spectrin mutation L253P associated with spinocerebellar ataxia type 5 interferes with binding to Arp1 and protein trafficking from the Golgi. Hum. Mol. Genet..

[4] Ikeda Y., Dick K.A., Weatherspoon M.R., Gincel D., Armbrust K.R., Dalton J.C. (2006). Spectrin mutations cause spinocerebellar ataxia type 5. Nat. Genet..

[5] Avery A.W., Crain J., Thomas D.D., Hays T.S. (2016). A human beta-III-spectrin spinocerebellar ataxia type 5 mutation causes high-affinity F-actin binding. Sci. Rep..

[6] Avery A.W., Fealey M.E., Wang F., Orlova A., Thompson A.R., Thomas D.D. (2017). Structural basis for high-affinity actin binding revealed by a beta-III-spectrin SCA5 missense mutation. Nat. Commun..

[7] Fujishima K., Kurisu J., Yamada M., Kengaku M. (2020). betaIII spectrin controls the planarity of Purkinje cell dendrites by modulating perpendicular axon-dendrite interactions. Development.

[8] Avery A.W., Thomas D.D., Hays T.S. (2017). beta-III-spectrin spinocerebellar ataxia type 5 mutation reveals a dominant cytoskeletal mechanism that underlies dendritic arborization. Proc. Natl. Acad. Sci. U. S. A..

[9] Armbrust K.R., Wang X., Hathorn T.J., Cramer S.W., Chen G., Zu T. (2014). Mutant beta-III spectrin causes mGluR1alpha mislocalization and functional deficits in a mouse model of spinocerebellar ataxia type 5. J. Neurosci..

[10] Gao Y., Perkins E.M., Clarkson Y.L., Tobia S., Lyndon A.R., Jackson M. (2011). beta-III spectrin is critical for development of purkinje cell dendritic tree and spine morphogenesis. J. Neurosci..

[11] Nicita F., Nardella M., Bellacchio E., Alfieri P., Terrone G., Piccini G. (2019). Heterozygous missense variants of SPTBN2 are a frequent cause of congenital cerebellar ataxia. Clin. Genet..

[12] Rebbeck R.T., Andrick A.K., Denha S.A., Svensson B., Guhathakurta P., Thomas D.D. (2021). Novel drug discovery platform for spinocerebellar ataxia, using fluorescence technology targeting beta-III-spectrin. J. Biol. Chem..

[13] Zhang J.H., Chung T.D., Oldenburg K.R. (1999). A Simple statistical parameter for use in evaluation and validation of high throughput screening assays. J. Biomol. Screen.

[14] Schaaf T.M., Li A., Grant B.D., Peterson K., Yuen S., Bawaskar P. (2018). Red-shifted FRET biosensors for high-throughput fluorescence lifetime screening. Biosensors (Basel).

[15] Schaaf T.M., Peterson K.C., Grant B.D., Bawaskar P., Yuen S., Li J. (2017). High-throughput spectral and lifetime-based FRET screening in living cells to identify small-molecule effectors of SERCA. SLAS Discov..

[16] Stroik D.R., Yuen S.L., Janicek K.A., Schaaf T.M., Li J., Ceholski D.K. (2018). Targeting protein-protein interactions for therapeutic discovery *via* FRET-based high-throughput screening in living cells. Sci. Rep..

[17] Gruber S.J., Cornea R.L., Li J., Peterson K.C., Schaaf T.M., Gillispie G.D. (2014). Discovery of enzyme modulators *via* high-throughput time-resolved FRET in living cells. J. Biomol. Screen.

[18] Rebbeck R.T., Essawy M.M., Nitu F.R., Grant B.D., Gillispie G.D., Thomas D.D. (2017). High-throughput screens to discover small-molecule modulators of ryanodine receptor calcium release channels. SLAS Discov..

[19] Rebbeck R.T., Singh D.P., Janicek K.A., Bers D.M., Thomas D.D., Launikonis B.S. (2020). RyR1-targeted drug discovery pipeline integrating FRET-based high-throughput screening and human myofiber dynamic Ca(2+) assays. Sci. Rep..

[20] Guhathakurta P., Prochniewicz E., Grant B.D., Peterson K.C., Thomas D.D. (2018). High-throughput screen, using time-resolved FRET, yields actin-binding compounds that modulate actin-myosin structure and function. J. Biol. Chem..

[21] Bunch T.A., Guhathakurta P., Lepak V.C., Thompson A.R., Kanassatega R.S., Wilson A. (2021). Cardiac myosin-binding protein C interaction with actin is inhibited by compounds identified in a high-throughput fluorescence lifetime screen. J. Biol. Chem..

[22] Lo C.H., Schaaf T.M., Grant B.D., Lim C.K., Bawaskar P., Aldrich C.C. (2019). Noncompetitive inhibitors of TNFR1 probe conformational activation states. Sci. Signal..

[23] Lo C.H., Vunnam N., Lewis A.K., Chiu T.L., Brummel B.E., Schaaf T.M. (2017). An innovative high-throughput screening approach for discovery of small molecules that inhibit TNF receptors. SLAS Discov..

[24] Lo C.H., Lim C.K., Ding Z., Wickramasinghe S.P., Braun A.R., Ashe K.H. (2019). Targeting the ensemble of heterogeneous tau oligomers in cells: a novel small molecule screening platform for tauopathies. Alzheimers Dement..

[25] Schaaf T.M., Peterson K.C., Grant B.D., Thomas D.D., Gillispie G.D. (2017). Spectral unmixing plate reader: high-throughput, high-precision FRET assays in living cells. SLAS Discov..

[26] Hughes J.P., Rees S., Kalindjian S.B., Philpott K.L. (2011). Principles of early drug discovery. Br. J. Pharmacol..

[27] Irwin J.J., Duan D., Torosyan H., Doak A.K., Ziebart K.T., Sterling T. (2015). An aggregation advisor for ligand discovery. J. Med. Chem..

[28] Weins A., Kenlan P., Herbert S., Le T.C., Villegas I., Kaplan B.S. (2005). Mutational and Biological Analysis of alpha-actinin-4 in focal segmental glomerulosclerosis. J. Am. Soc. Nephrol..

[29] Duff R.M., Tay V., Hackman P., Ravenscroft G., McLean C., Kennedy P. (2011). Mutations in the N-terminal actin-binding domain of filamin C cause a distal myopathy. Am. J. Hum. Genet..

[30] Wasmann R.E., Muilwijk E.W., Burger D.M., Verweij P.E., Knibbe C.A., Bruggemann R.J. (2018). Clinical pharmacokinetics and pharmacodynamics of micafungin. Clin. Pharmacokinet..

[31] Marena G.D., Dos Santos Ramos M.A., Bauab T.M., Chorilli M. (2021). Biological properties and analytical methods for micafungin: a critical review. Crit. Rev. Anal. Chem..

[32] Felton T., Troke P.F., Hope W.W. (2014). Tissue penetration of antifungal agents. Clin. Microbiol. Rev..

[33] Ahmed T., Raza S.H., Maryam A., Setzer W.N., Braidy N., Nabavi S.F. (2016). Ginsenoside Rb1 as a neuroprotective agent: a review. Brain Res. Bull..

[34] Zhang Y.L., Liu Y., Kang X.P., Dou C.Y., Zhuo R.G., Huang S.Q. (2018). Ginsenoside Rb1 confers neuroprotection *via* promotion of glutamate transporters in a mouse model of Parkinson's disease. Neuropharmacology.

[35] Perkins E.M., Clarkson Y.L., Sabatier N., Longhurst D.M., Millward C.P., Jack J. (2010). Loss of beta-III spectrin leads to Purkinje cell dysfunction recapitulating the behavior and neuropathology of spinocerebellar ataxia type 5 in humans. J. Neurosci..

[36] Perkins E.M., Suminaite D., Clarkson Y.L., Lee S.K., Lyndon A.R., Rothstein J.D. (2016). Posterior cerebellar Purkinje cells in an SCA5/SPARCA1 mouse model are especially vulnerable to the synergistic effect of loss of beta-III spectrin and GLAST. Hum. Mol. Genet..

[37] Denha S.A., Atang A.E., Hays T.S., Avery A.W. (2022). beta-III-spectrin N-terminus is required for high-affinity actin binding and SCA5 neurotoxicity. Sci. Rep..

[38] Guhathakurta P., Prochniewicz E., Thomas D.D. (2015). Amplitude of the actomyosin power stroke depends strongly on the isoform of the myosin essential light chain. Proc. Natl. Acad. Sci. U. S. A.

[39] Muretta J.M., Kyrychenko A., Ladokhin A.S., Kast D., Gillispie G.E., Thomas D.D. (2010). High -performance time-resolved fluorescence by direct waveform recording. Rev. Sci. Instrum.

[40] Schaaf T.M., Kleinboehl E., Yuen S.L., Roelike L.N., Svensson B., Thompson A.R. (2020). Live-cell cardiac-specific high-throughput screening platform for drug-like molecules that enhance Ca(2+) transport. Cells.

[41] Humphrey W., Dalke A., Schulten K. (1996). Vmd: visual molecular dynamics. J. Mol. Graph.

