# Supplementary material for: Early-phase drug discovery of β-III-spectrin actin-binding modulators for treatment of spinocerebellar ataxia type 5
Source: J Biol Chem. 2023 Jan 31;299(3):102956. doi: 10.1016/j.jbc.2023.102956 (PMC9978034; doi:10.1016/j.jbc.2023.102956)
Supplement: Supplementary figure [file mmc1.pdf]

Supporting Information

Early phase drug discovery of β-III-spectrin actin-binding modulators for treatment of spinocerebellar ataxia type 5

Piyali Guhathakurta[1#], Robyn T. Rebbeck[1#], Sarah A. Denha[2], Amanda R. Keller[2], Anna L. Carter[1], Alexandra E. Atang[2], Bengt Svensson[1], David D. Thomas[1], Thomas S. Hays[3] and Adam W. Avery[2*]

[1] Department of Biochemistry, Molecular Biology and Biophysics, University of Minnesota, Minneapolis, MN 55455, USA;
[2] Department of Chemistry, Oakland University, Rochester, MI 48309-4479, USA;
[3] Department of Genetics, Cellular Biology, and Development, University of Minnesota, Minneapolis, MN 55455, USA;

[#] equal contribution to the manuscript
***** Corresponding Author: Adam W. Avery
Email: awavery@oakland.edu

Running title: ***In vitro* drug discovery targeting β-III-spectrin**

Table S1. Aggregator Database results for Hit compounds. Many of the Hits that we determined experimentally to be ABD aggregators are either known aggregators or somewhat/very similar to known aggregators. In contrast, Hits that did not cause ABD aggregation are not similar to known aggregators. High logP indicates compound is hydrophobic and has increased potential to act as aggregator.

Hit	Known Aggregator?
Candesartan	Very similar to known aggregator
Oleic acid	Very similar to known aggregator
Docusate	Not similar to known aggregator, but high logP
Zafirlukast	Very similar to known aggregator
Montelukast	Not similar to known aggregator, but high logP
Closantel	Data not available
Moxidectin	Not similar to known aggregator, but high logP
Micafungin	Not similar to known aggregator
Quinacrine	Not similar to known aggregator, but high logP
Tacrolimus	Not similar to known aggregator, but high logP
Lovastatin	Not similar to known aggregator, but high logP
Sclareol	Not similar to known aggregator, but high logP
Ginsenoside Rb1	Not similar to known aggregator
Cabazitaxel	Not similar to known aggregator, but high logP
Ascomycin	Not similar to known aggregator, but high logP
Avanafil	Not similar to known aggregator, but high logP
Piroctone	Not similar to known aggregator
Bosetan	Very similar to known aggregator

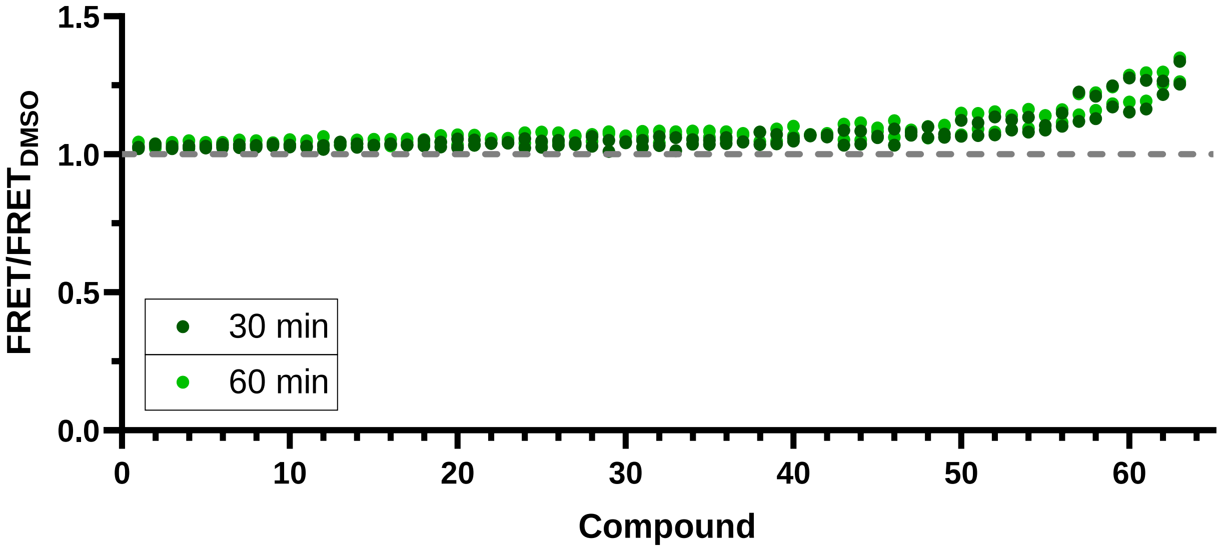

Figure S1. Hits that reproducibly increased *in vitro* ABD biosensor FRET between two screens of the Selleck library. Relative FRET effect of Selleck Hits that were identified (with 5SD threshold) increasing FRET in both screen runs. Data is shown as relative to DMSO control (gray dotted line). Chemical names and structures shown in Figures S2 and 11-16.

1	Piperonyl butoxide
2	Dipyridamole
3	**Candesartan Cilexetil**
4	**Oleic Acid**
5	**Micafungin Sodium**
6	**Docusate Sodium**
7	**Montelukast Sodium**
8	Sodium lauryl sulfate
9	**Quinacrine 2HCl**
10	Quinacrine 2HCl 2H2O
11	**Zafirlukast**
12	Dalbavancin
13	Erythromycin estolate
14	Tyrosol
15	Cefsulodin sodium
16	Ledipasvir (GS5885)
17	**Temsirolimus**
18	Thimerosal
19	Montelukast
20	Zotarolimus(ABT-578)
21	**Ombitasvir (ABT-267)**
22	**Batyl alcohol**
23	**cis-Anethole**
24	Bronopol
25	Anidulafungin (LY303366)
26	Simvastatin
27	Ridaforolimus (Deforolimus)
28	**Moxidectin**
29	Aprepitant
30	Asunaprevir
31	Rapamycin (Sirolimus)
32	Simeprevir
33	Manidipine 2HCl
34	Clindamycin palmitate HCl
35	Ertapenem sodium
36	**Ethotoin**
37	Hydroxyprogesterone caproate
38	Latanoprost
39	Cilnidipine
40	Ouabain
41	**Carfilzomib**
42	Dabigatran Etexilate
43	Thiostrepton
44	Rolapitant
45	Daclatasvir (BMS-790052)
46	Celecoxib
47	Tafluprost
48	**Vorapaxar**
49	Grazoprevir
50	Ibuprofen piconol
51	**Cabazitaxel**
52	**Ivermectin**
53	**Avermectin B1(Abamectin)**
54	**Tacrolimus (FK506)**
55	**Clonidine HCl**
56	Troglitazone (CS-045)
57	**cholecalciferol**
58	Nefazodone hydrochloride
59	**Laurocapram**
60	Cabozantinib malate (XL184)
61	Ketoconazole
62	Econazole
63	**Bosentan Hydrate**
64	**Avanafil**
65	**Miconazole Nitrate**
66	Obeticholic Acid
67	**Ascomycin (FK520)**
68	Everolimus (RAD001)
69	**Ginsenoside Rb1**
70	**Amorolfine HCl**
71	Cobicistat (GS-9350)
72	Luteolin
73	Ampicillin Trihydrate
74	**Nilotinib hydrochloride**
75	**Securinine**
76	**Sclareol**
77	Pneumocandin B0
78	**AKBA**
79	Calcitriol
80	**Hydroxyzine pamoate**
81	Saikosaponin A
82	Canagliflozin hemihydrate
83	Efonidipine
84	**Avatrombopag**
85	**Lapatinib Ditosylate**
86	**Lovastatin**
87	**Piroctone Olamine**
88	Ceftibuten dihydrate
89	**Closantel**
90	Fursultiamine
91	Triflusal
92	Succimer
93	Bromocriptine Mesylate
94	Cisatracurium Besylate
95	Amsacrine hydrochloride
96	Clomipramine HCl
97	Primaquine Diphosphate
98	Salmeterol
99	Doxycycline Hyclate
100	Retigabine 2HCl
101	Fangchinoline
102	Silymarin
103	Sulbutiamine
104	Levosimendan
105	Olodaterol hydrochloride
106	Pimavanserin
107	Paroxetine mesylate
108	Paroxetine HCl
109	Nintedanib Ethanesulfonate Salt
110	Nifedipine
111	pyrvinium
112	Benzalkonium chloride
113	Carvedilol Phosphate
114	(S)-crizotinib
115	Flupirtine maleate
116	Tigecycline
117	Ilaprazole
118	Methylcobalamin
119	CP21R7 (CP21)
120	Sunitinib Malate
121	Nebivolol HCl
122	(−)-Norepinephrine
123	Crizotinib (PF-02341066)
124	Omadacycline tosylate
125	Anlotinib (AL3818) 2HCl
126	Meclocycline Sulfosalicylate
127	Trichlormethiazide
128	Vortioxetine HBr
129	Benzethonium Chloride
130	Cyclofenil
131	Mivacurium chloride
132	Raloxifene HCl
133	Tegaserod Maleate
134	Ethidium bromide
135	Ethacridine lactate monohydrate
136	Methylene Blue
137	Bacitracin Zinc
138	Phenazine methosulfate
139	Chlorhexidine 2HCl
140	Domiphen Bromide
141	Chlorhexidine
142	Sanguinarine chloride
143	Fingolimod (FTY720) HCl
144	4-Aminophenol
145	Zinc Undecylenate
146	Zinc Pyrithione
147	Otilonium Bromide
148	Olanexidine HCl semihydrate
149	Cetrimonium Bromide (CTAB)
150	Cetylpyridinium Chloride
151	Pixantrone Maleate
152	Octenidine Dihydrochloride

Figure S2. Compound names for Hits that altered *in vitro* ABD biosensor FRET, shown in Figures 3 and S1. Thirty eight Hits that were chosen for further testing are indicated by green text.

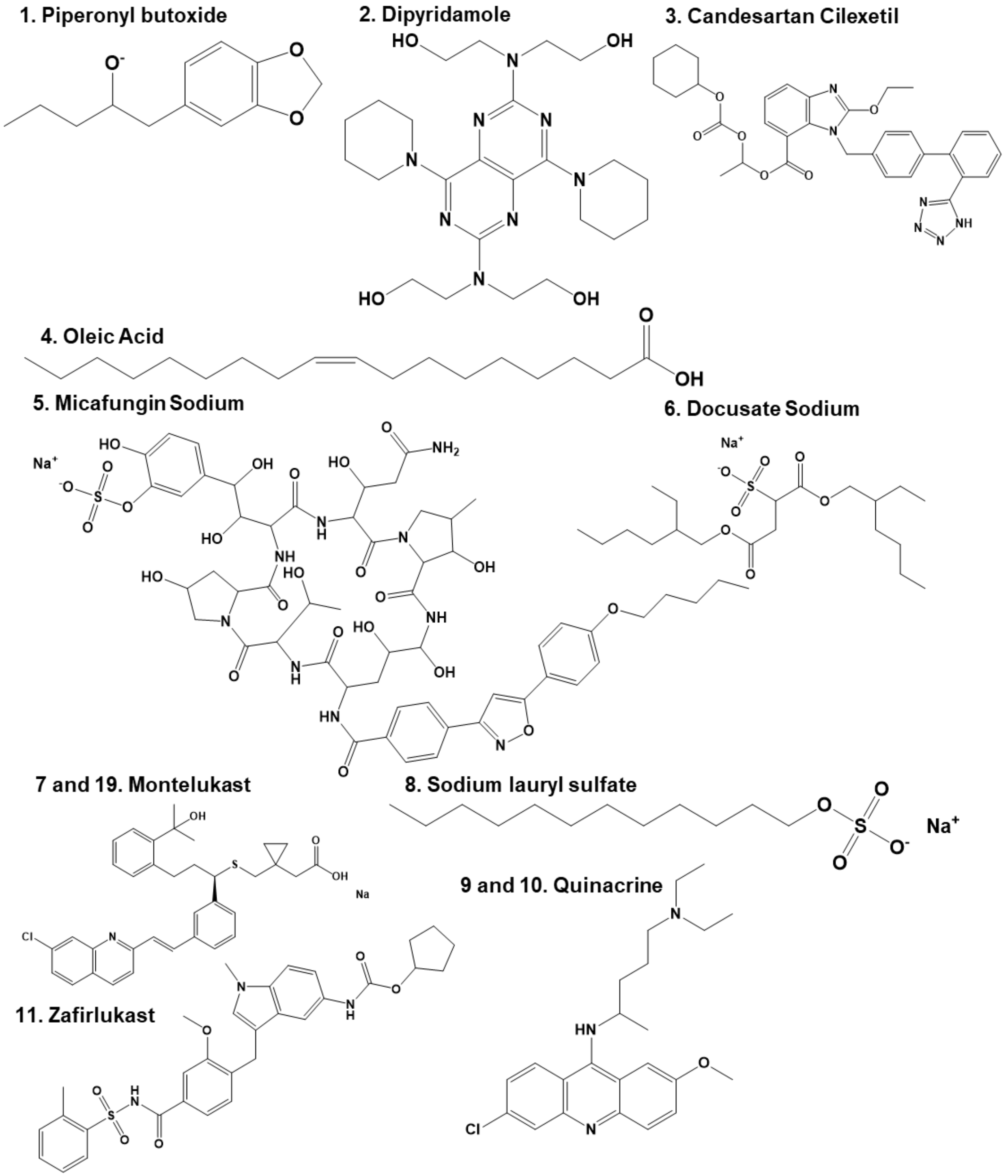

Figure S3. Chemical structures of Selleck screen Hits.

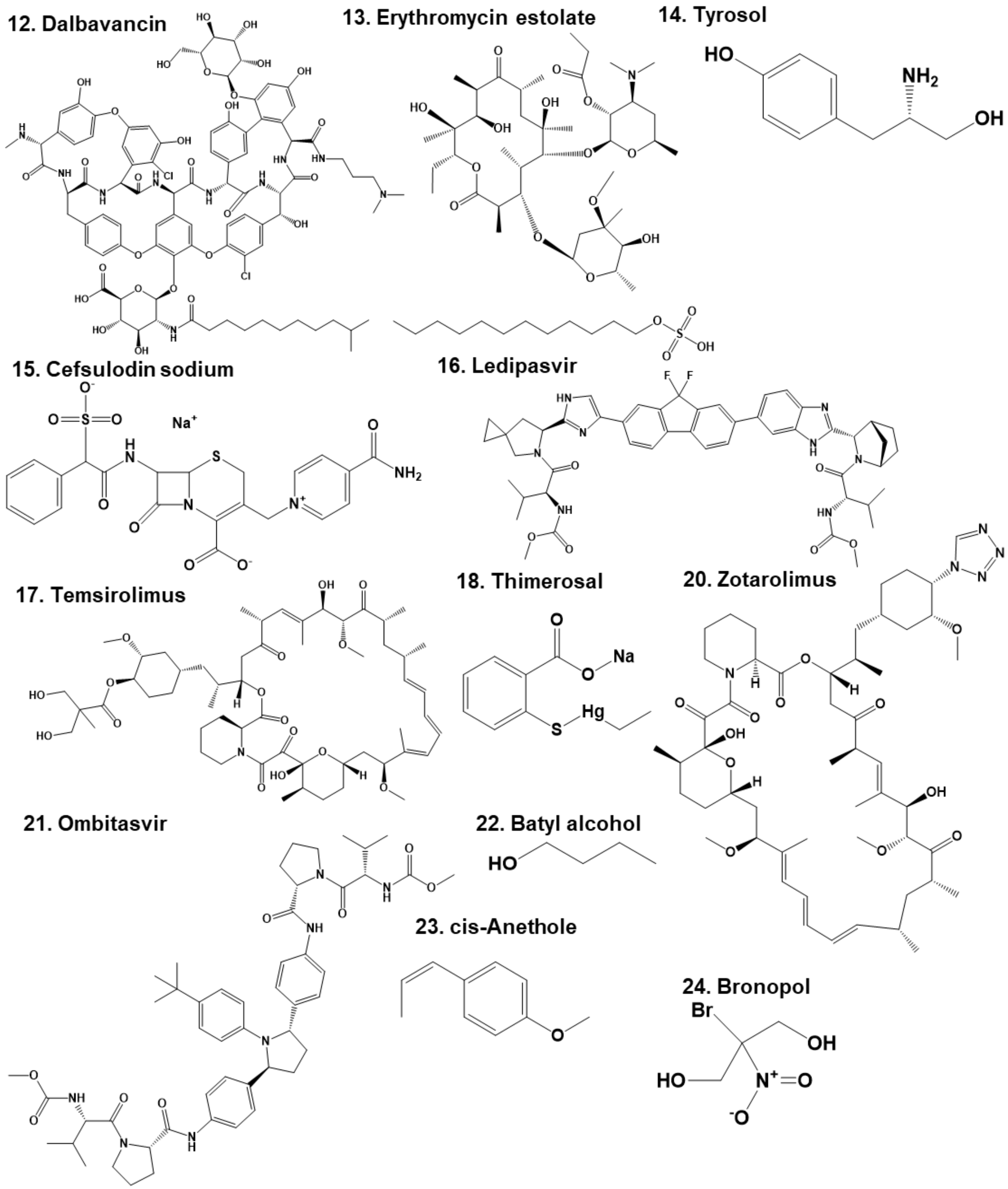

Figure S4. Chemical structures of Selleck screen Hits.

25. Anidulafungin

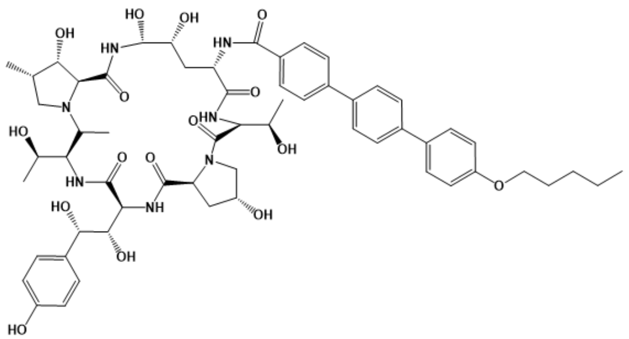

26. Simvastatin

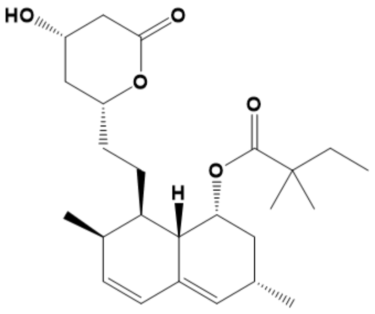

27. Ridaforolimus

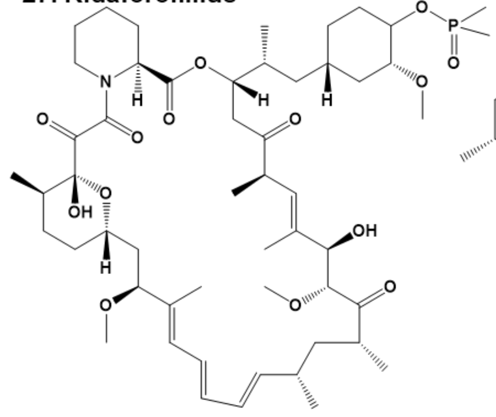

28. Moxidectin

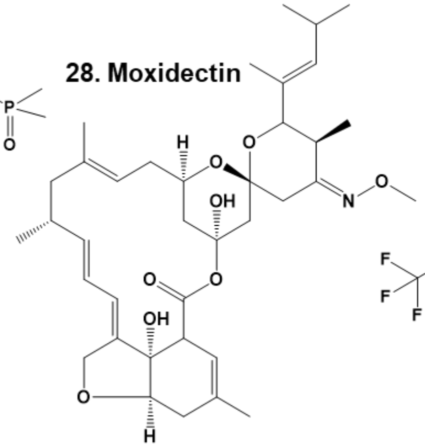

29. Aprepitant

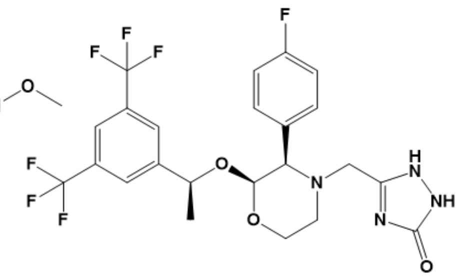

30. Asunaprevir

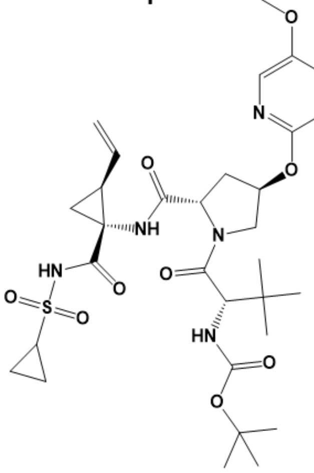

31. Rapamycin

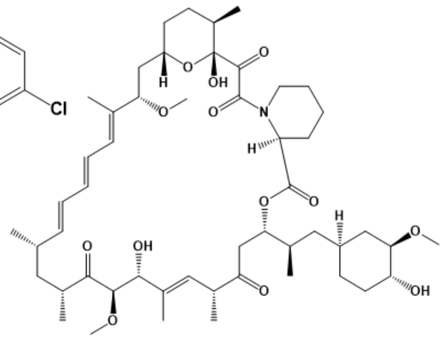

32. Simeprevir

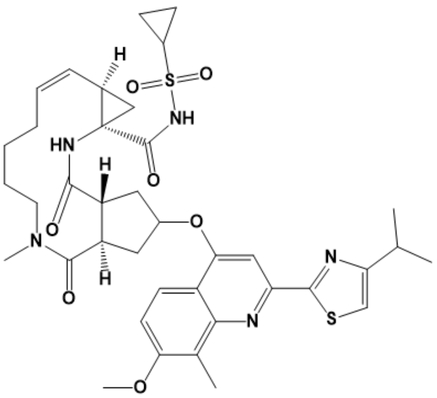

33. Manidipine

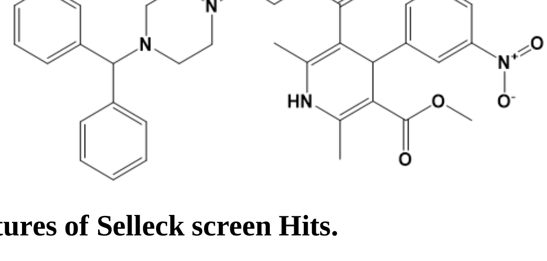

Figure S5. Chemical structures of Selleck screen Hits.

34. Clindamycin palmitate

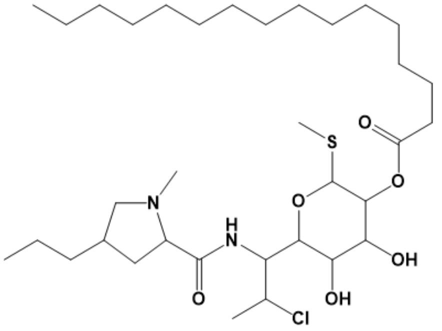

35. Ertapenem sodium

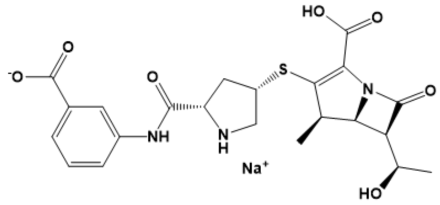

36. Ethotoin

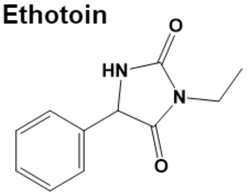

37. Hydroxyprogesterone caproate

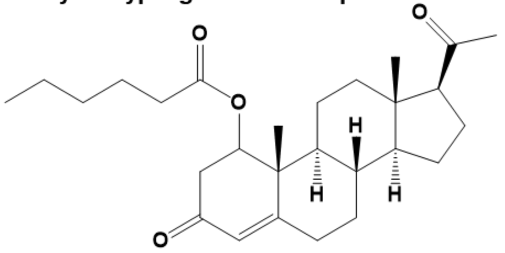

38. Latanoprost

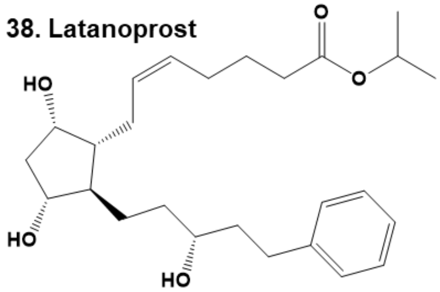

39. Cilnidipine

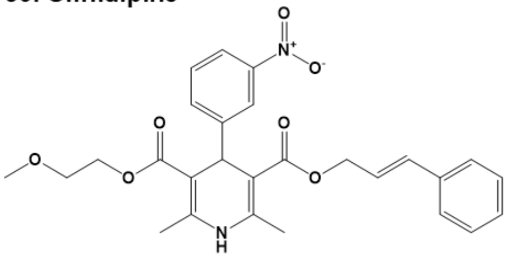

40. Ouabain

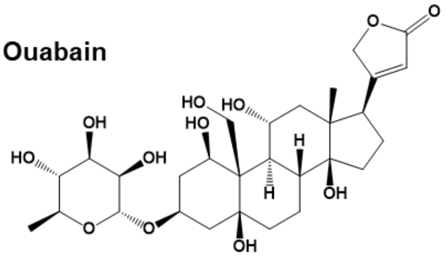

41. Carfilzomib

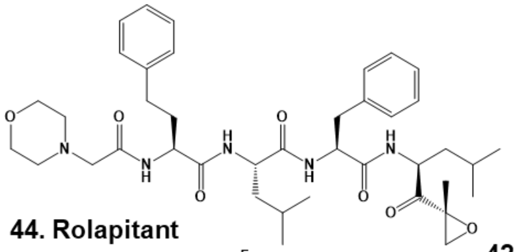

43. Thiostrepton

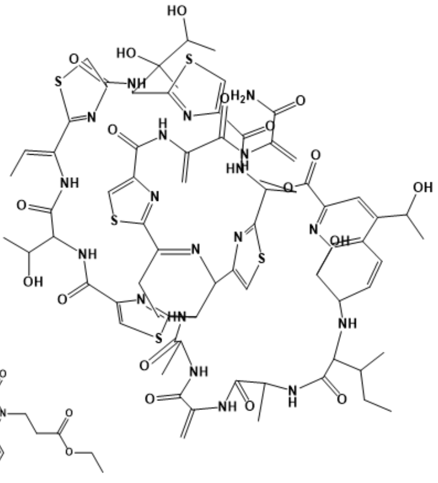

44. Rolapitant

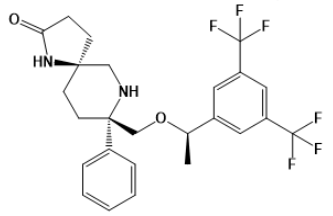

42. Dabigatran Etexilate

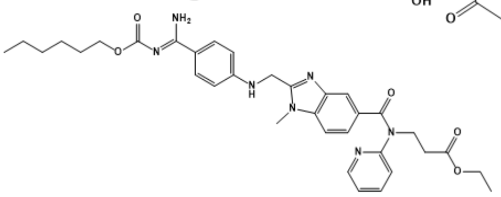

Figure S6. Chemical structures of Selleck screen Hits.

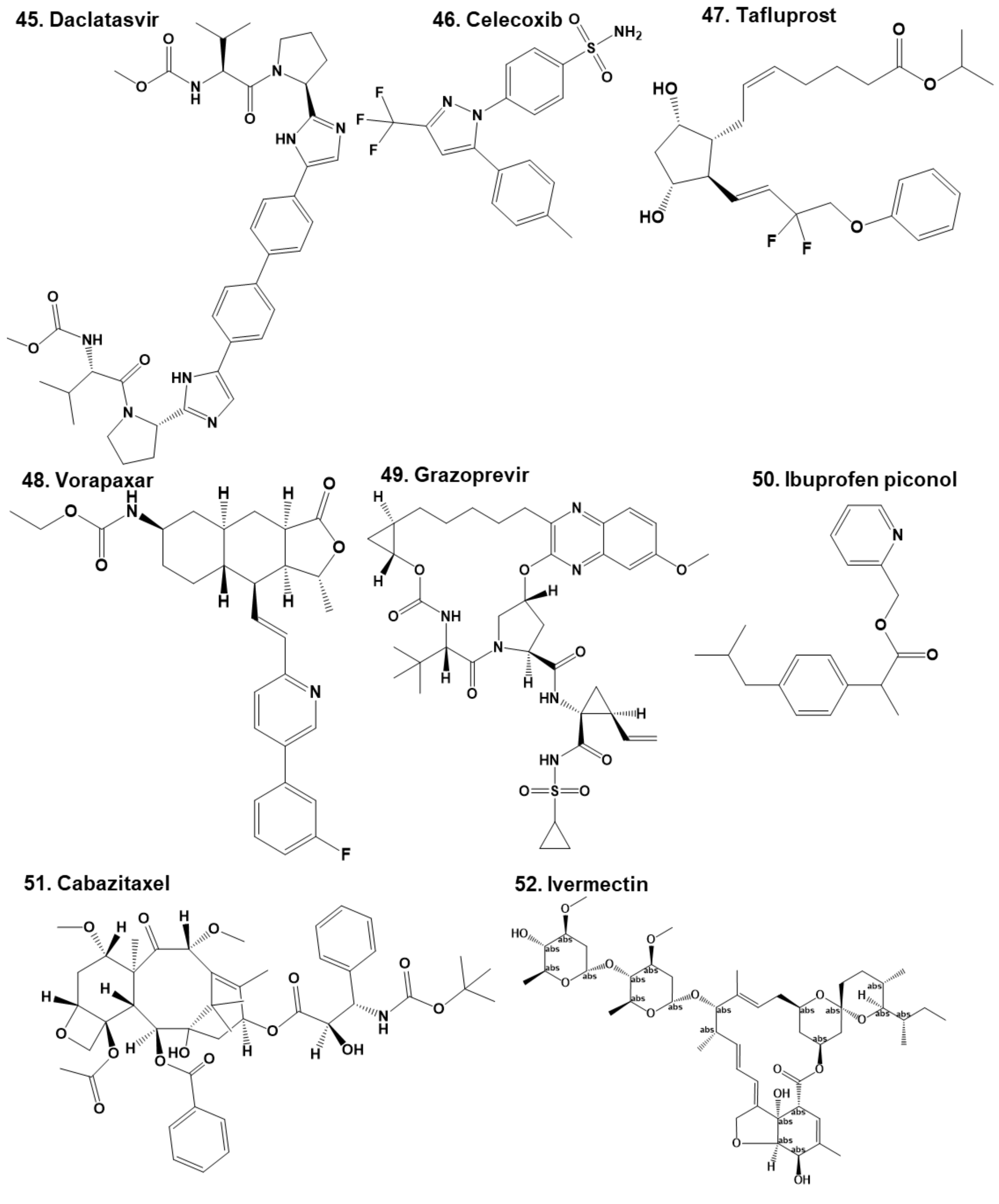

Figure S7. Chemical structures of Selleck screen Hits.

53. Avermectin B1

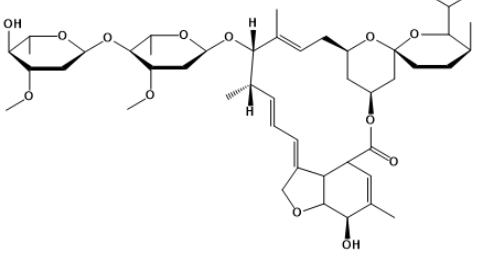

54. Tacrolimus

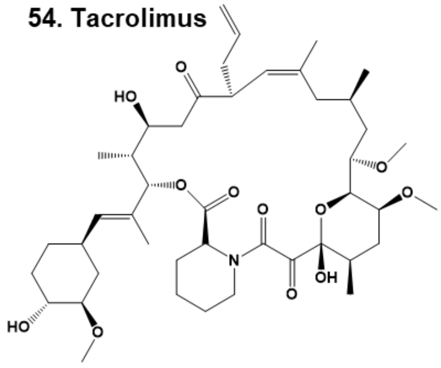

55. Clonidine

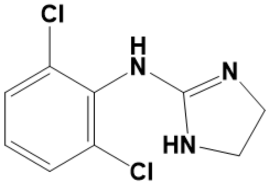

56. Troglitazone

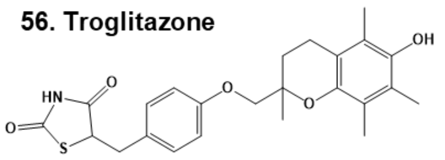

57. cholecalciferol

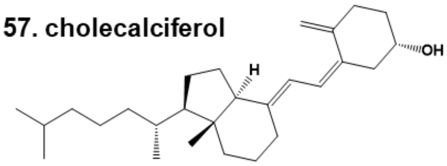

58. Nefazodone hydrochloride

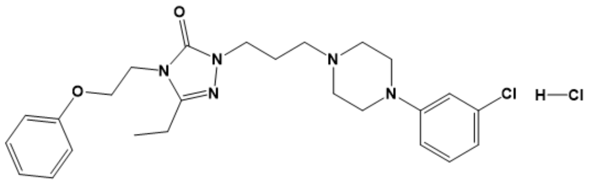

59. Laurocapram

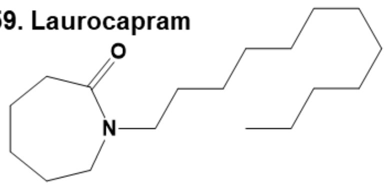

60. Cabozantinib malate

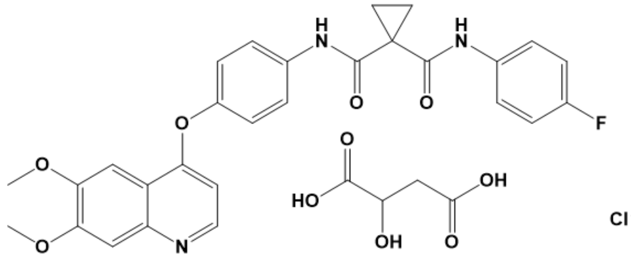

61. Ketoconazole

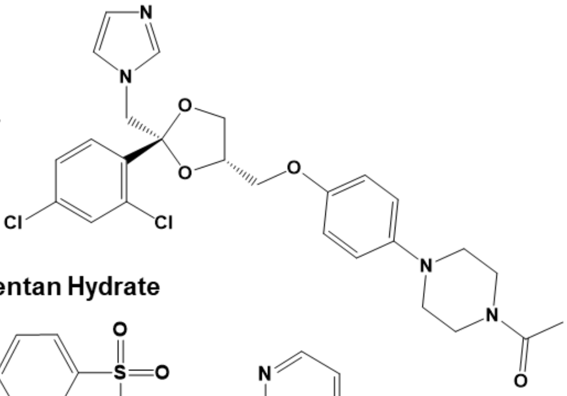

62. Econazole

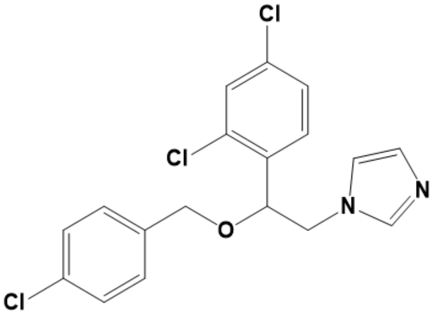

63. Bosentan Hydrate

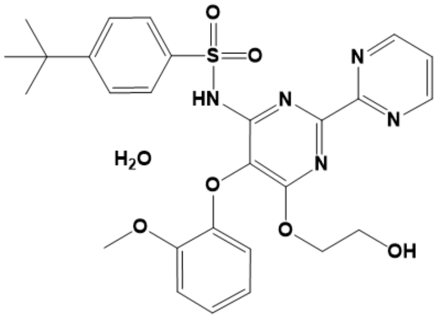

Figure S8. Chemical structures of Selleck screen Hits.

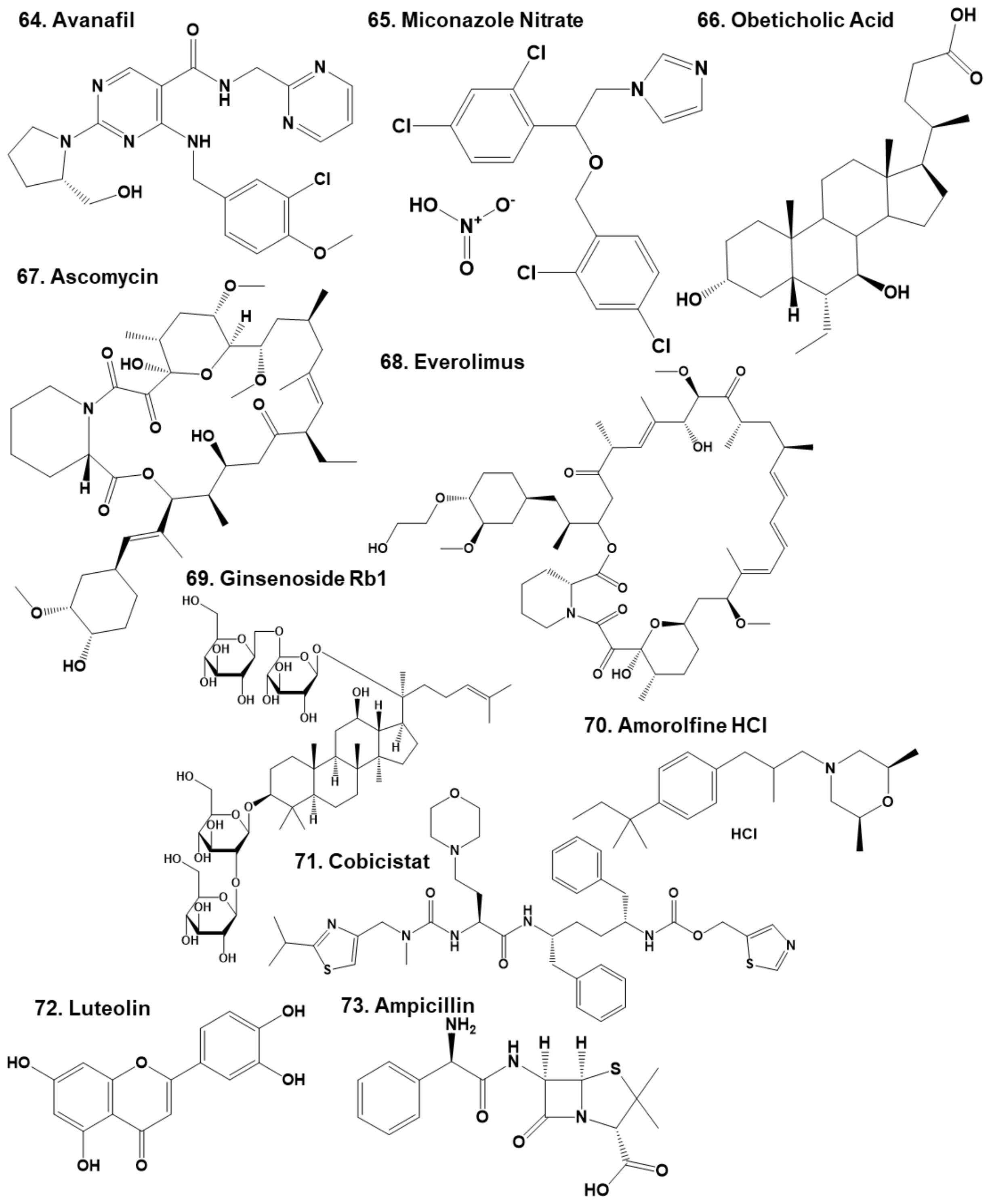

Figure S9. Chemical structures of Selleck screen Hits.

74. Nilotinib

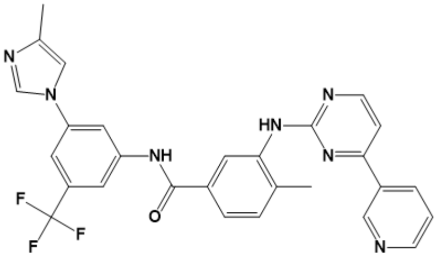

75. Securinine

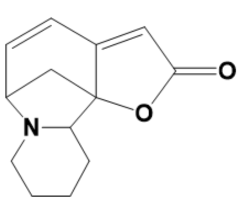

76. Sclareol

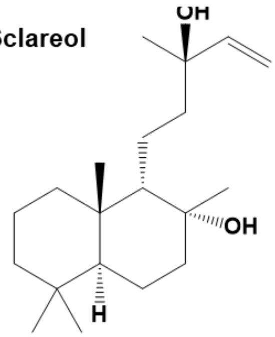

77. Pneumocandin B0

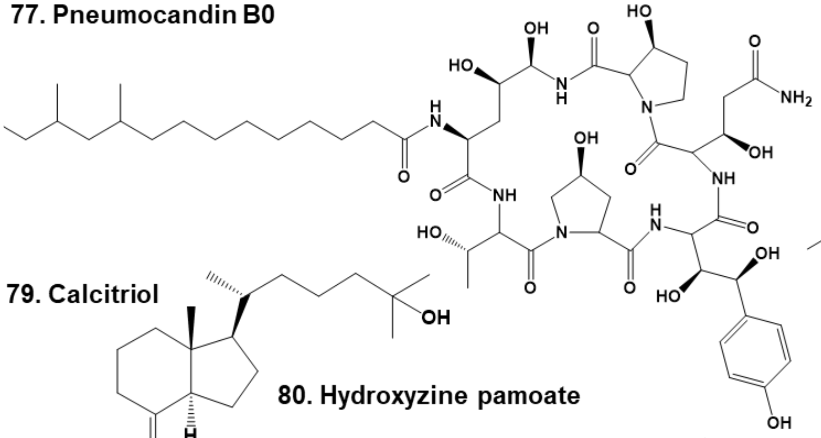

78. AKBA

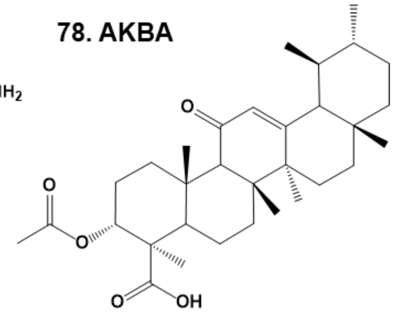

79. Calcitriol

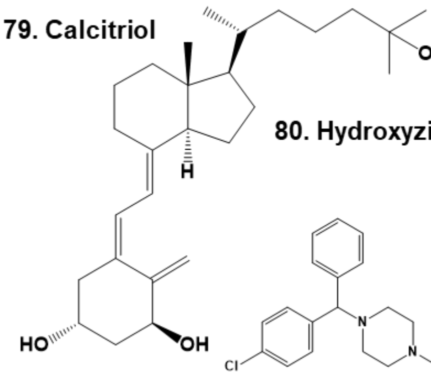

80. Hydroxyzine pamoate

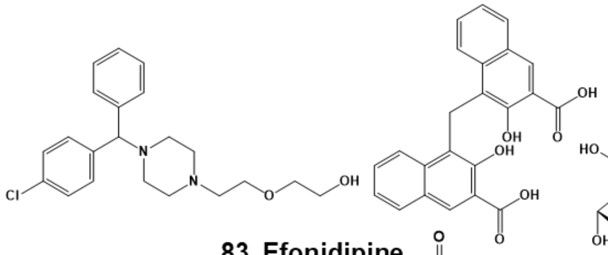

81. Saikosaponin A

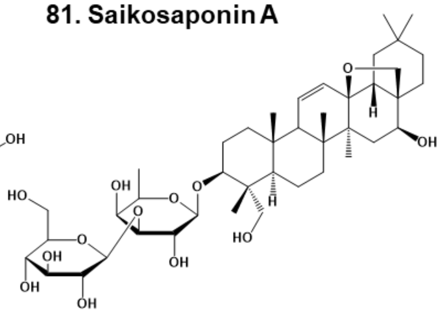

82. Canagliflozin

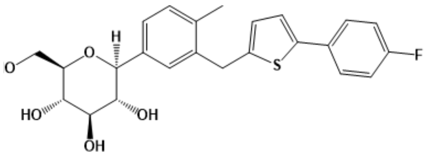

83. Efonidipine

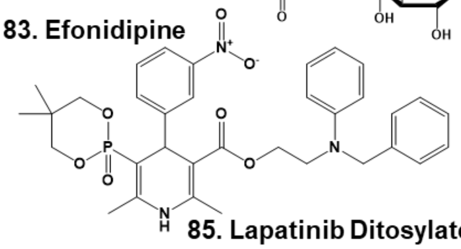

84. Avatrombopag

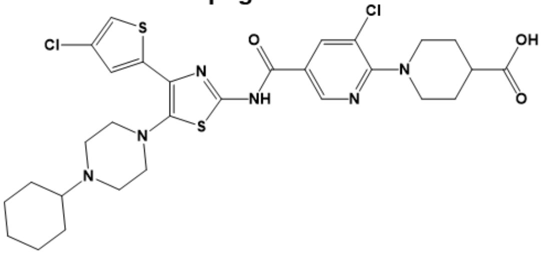

85. Lapatinib Ditosylate

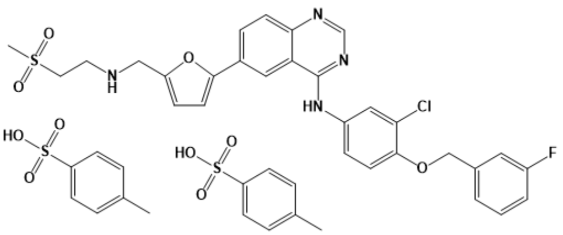

Figure S10. Chemical structures of Selleck screen Hits.

86. Lovastatin

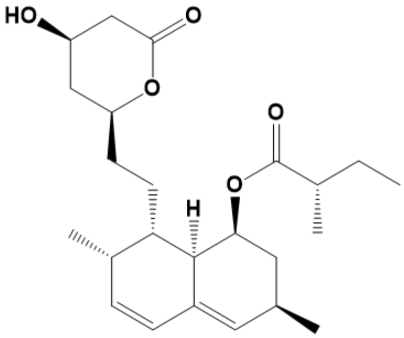

87. Piroctone Olamine

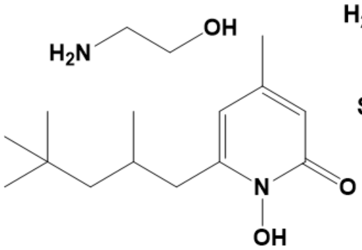

88. Ceftibuten dihydrate

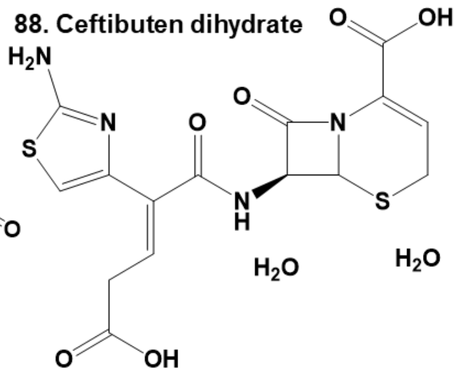

89. Closantel

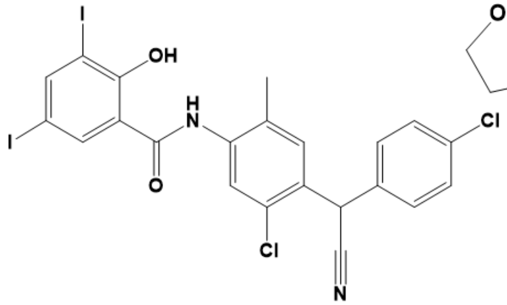

90. Fursultiamine

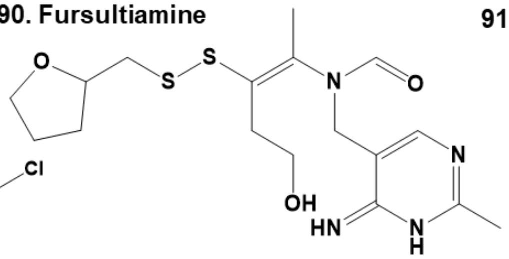

91. Triflusal

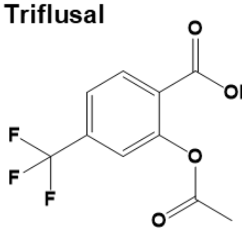

92. Succimer

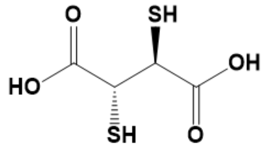

93. Bromocriptine Mesylate

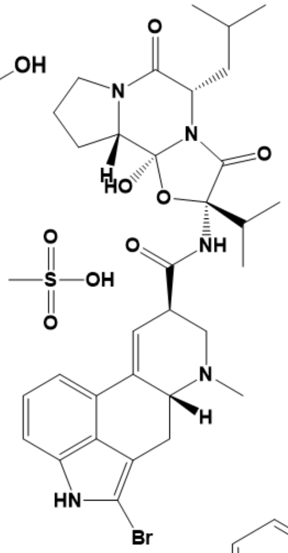

94. Cisatracurium Besylate

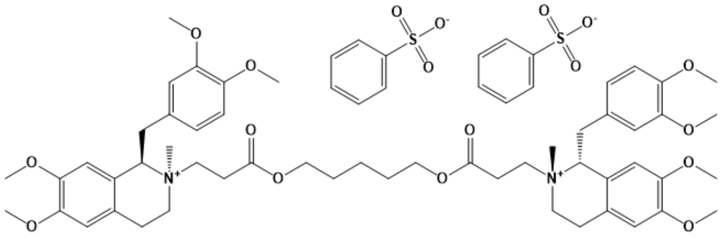

95. Amsacrine HCl

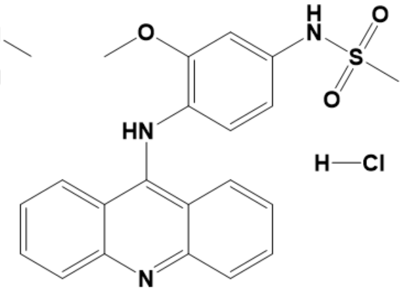

96. Clomipramine HCl

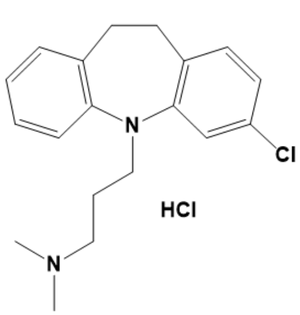

Figure S11. Chemical structures of Selleck screen Hits.

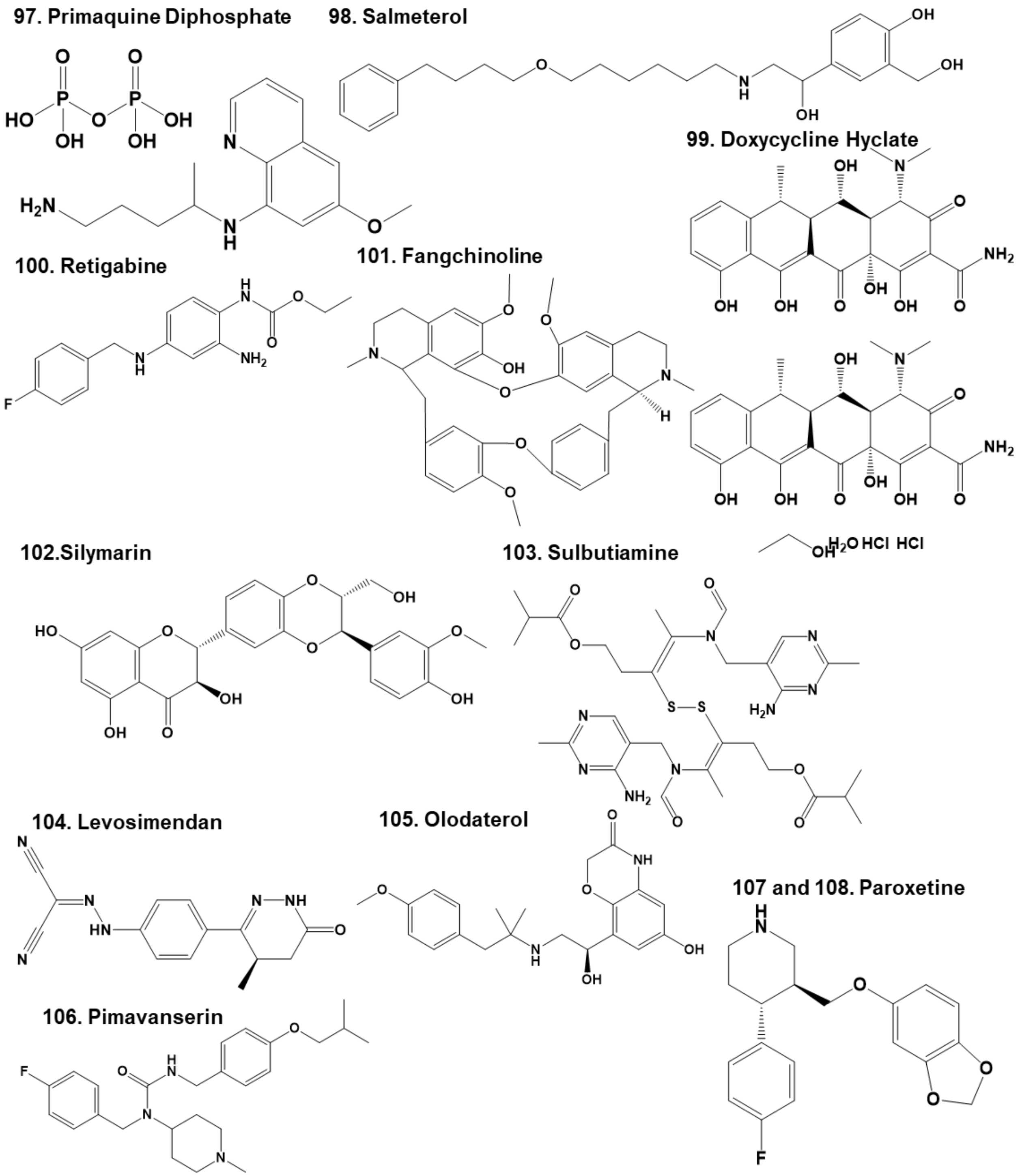

Figure S12. Chemical structures of Selleck screen Hits.

109. Nintedanib Ethanesulfonate

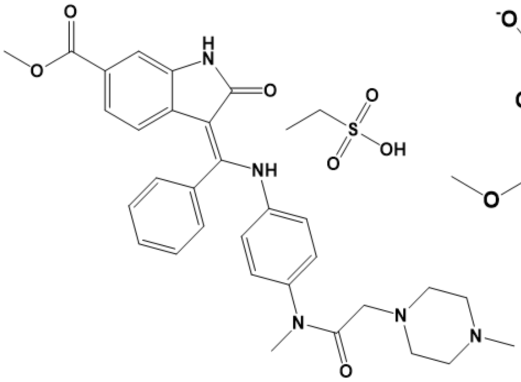

110. Nifedipine

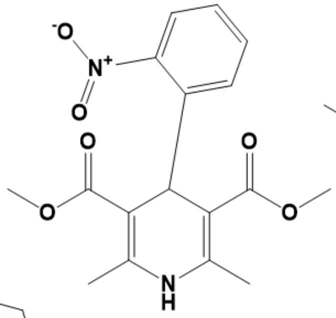

111. pyrvinium

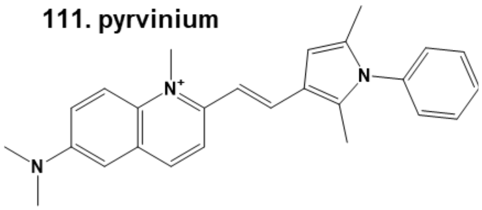

112. Benzalkonium chloride

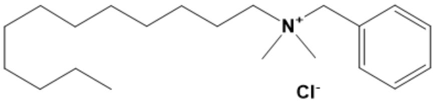

113. Carvedilol

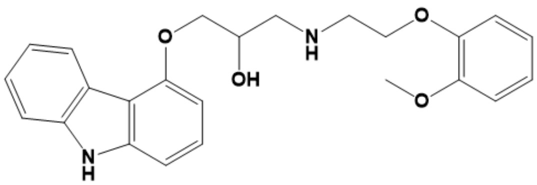

114. (S)-crizotinib

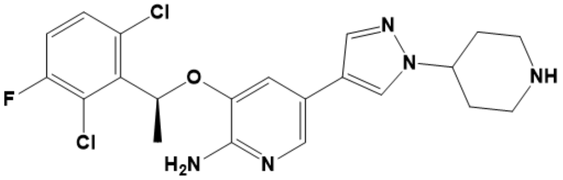

115. Flupirtine maleate

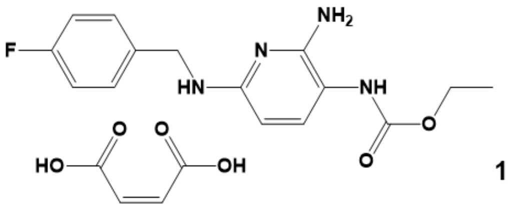

116. Tigecycline

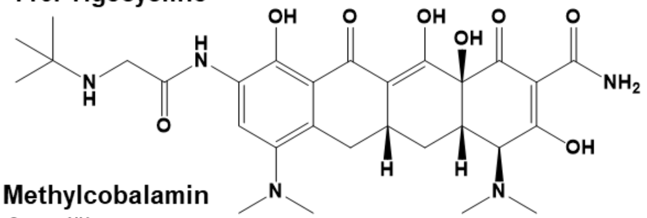

117. Ilaprazole

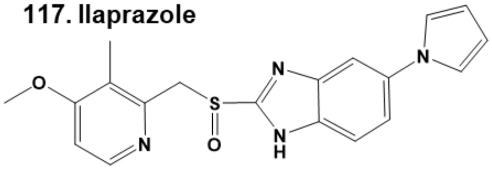

118. Methylcobalamin

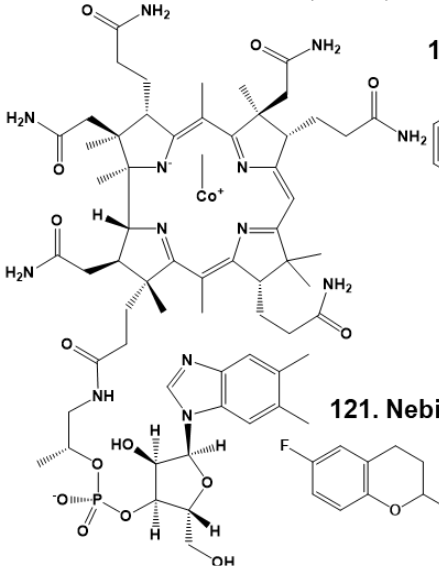

119. CP21R7 (CP21)

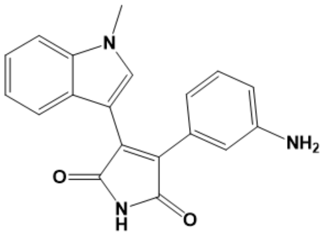

120. Sunitinib Malate

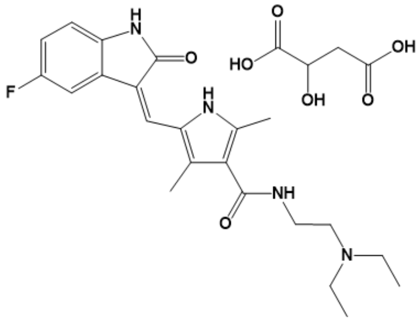

121. Nebivolol

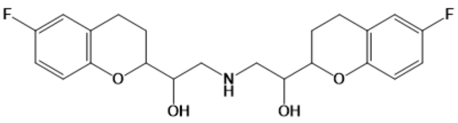

Figure S13. Chemical structures of Selleck screen Hits.

122. (−)-Norepinephrine

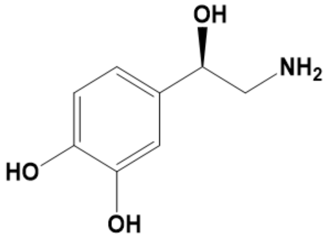

123. Crizotinib (PF-02341066)

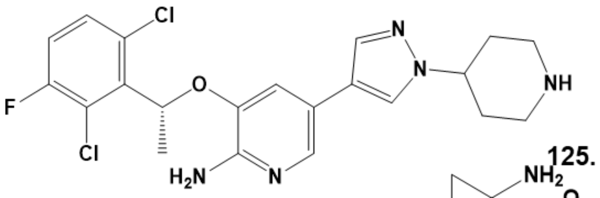

125. Anlotinib

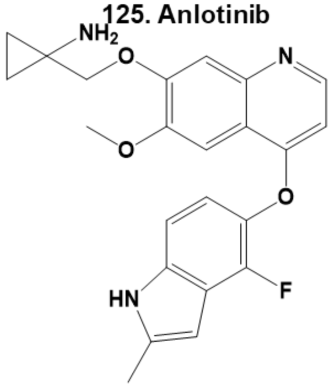

124. Omadacycline tosylate

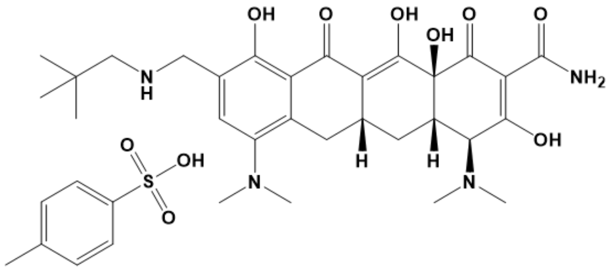

126. Meclocycline Sulfosalicylate

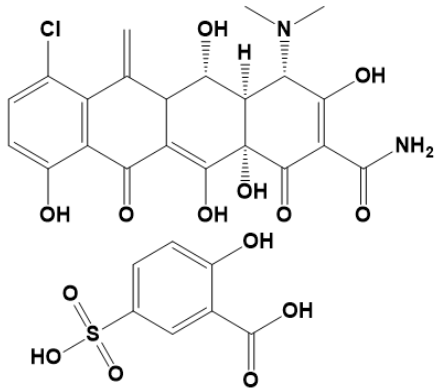

127. Trichlormethiazide

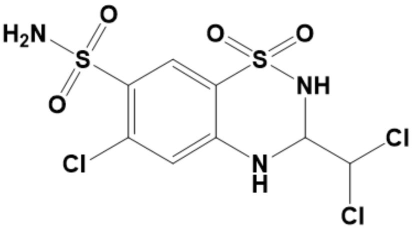

128. Vortioxetine (Lu AA21004)

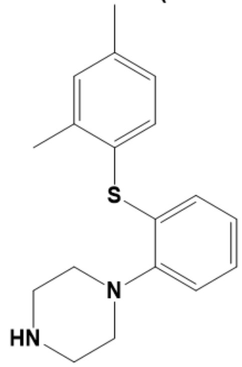

129. Benzethonium

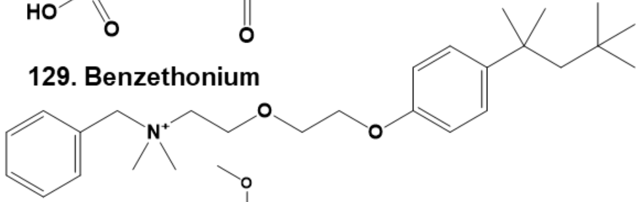

130. Cyclofenil

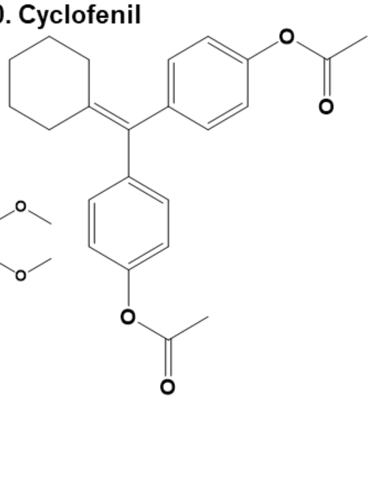

131. Mivacurium

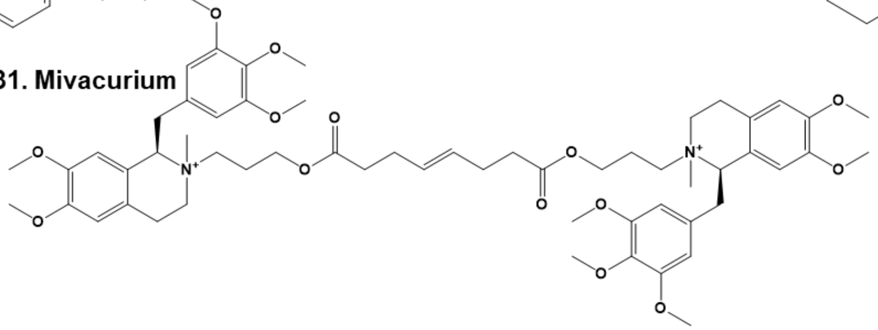

Figure S14. Chemical structures of Selleck screen Hits.

132. Raloxifene

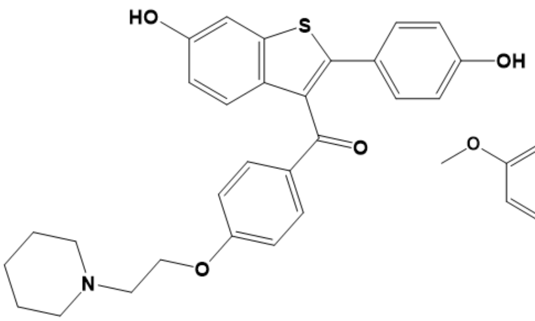

133. Tegaserod Maleate

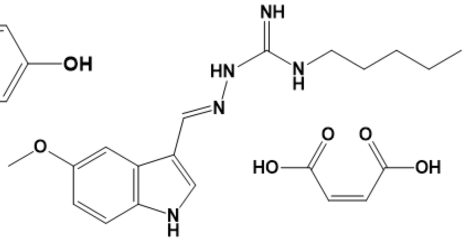

134. Ethidium bromide

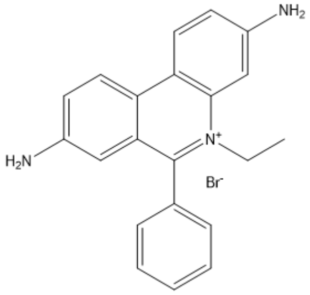

135. Ethacridine lactate monohydrate

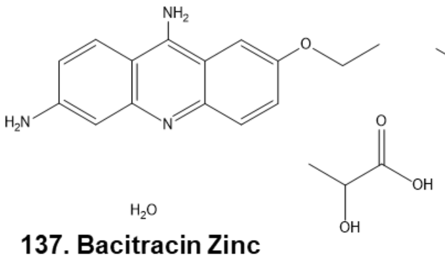

136. Methylene Blue

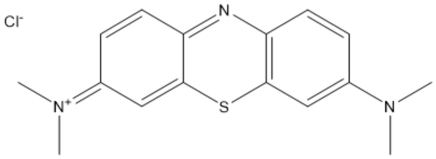

138. Phenazine methosulfate

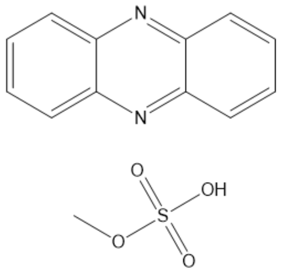

137. Bacitracin Zinc

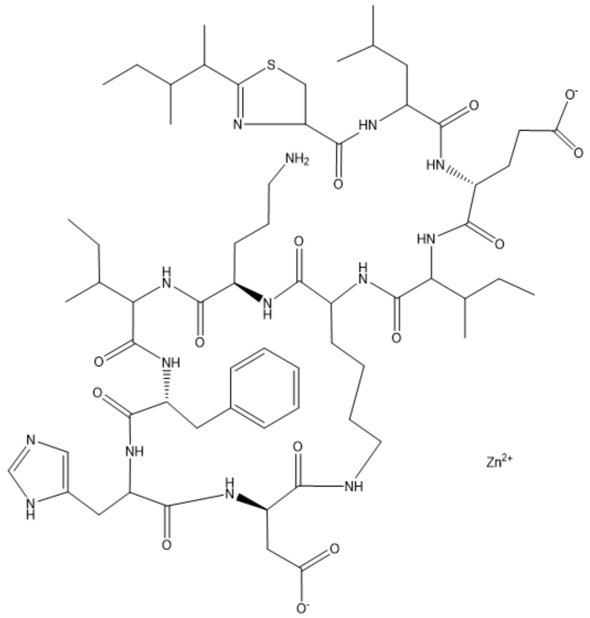

142. Sanguinarine chloride

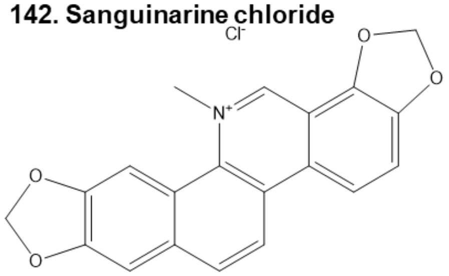

143. Fingolimod (FTY720) HCl

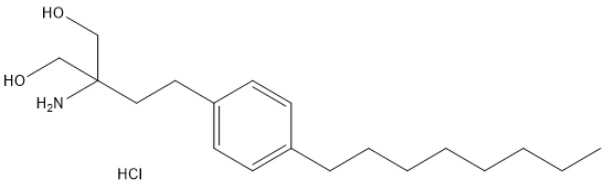

144. 4-Aminophenol

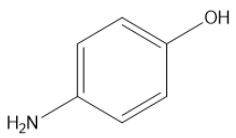

139 and 141. Chlorhexidine

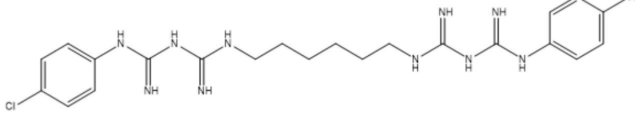

Figure S15. Chemical structures of Selleck screen Hits.

145. Zinc Undecylenate

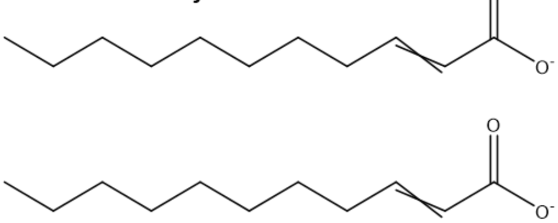

146. Zinc Pyrithione

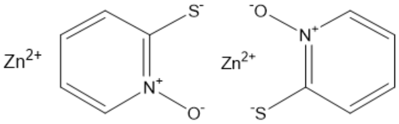

147. Otilonium Bromide

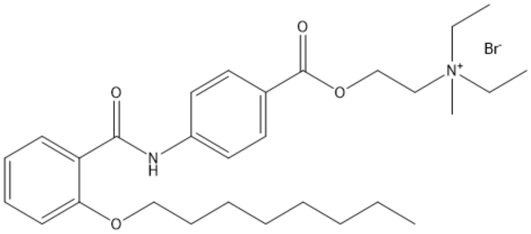

148. Olanexidine Hydrochloride semihydrate

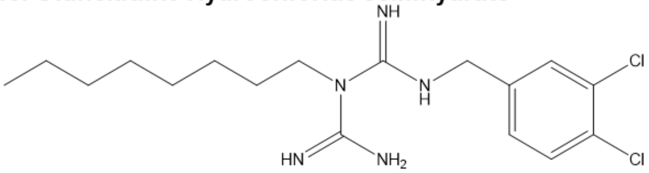

149. Cetrimonium Bromide

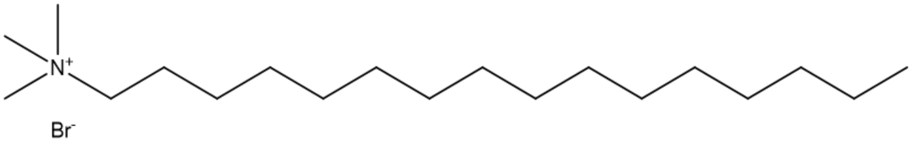

150. Cetylpyridinium Chloride

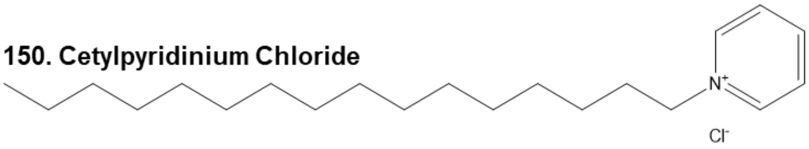

151. Pixantrone Maleate

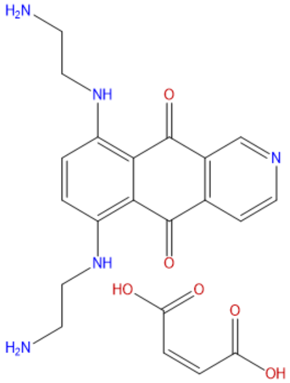

152. Octenidine Dihydrochloride

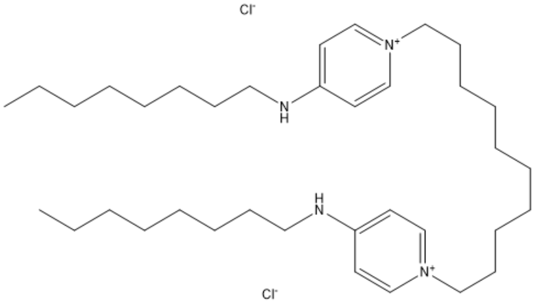

Figure S16. Chemical structures of Selleck screen Hits.

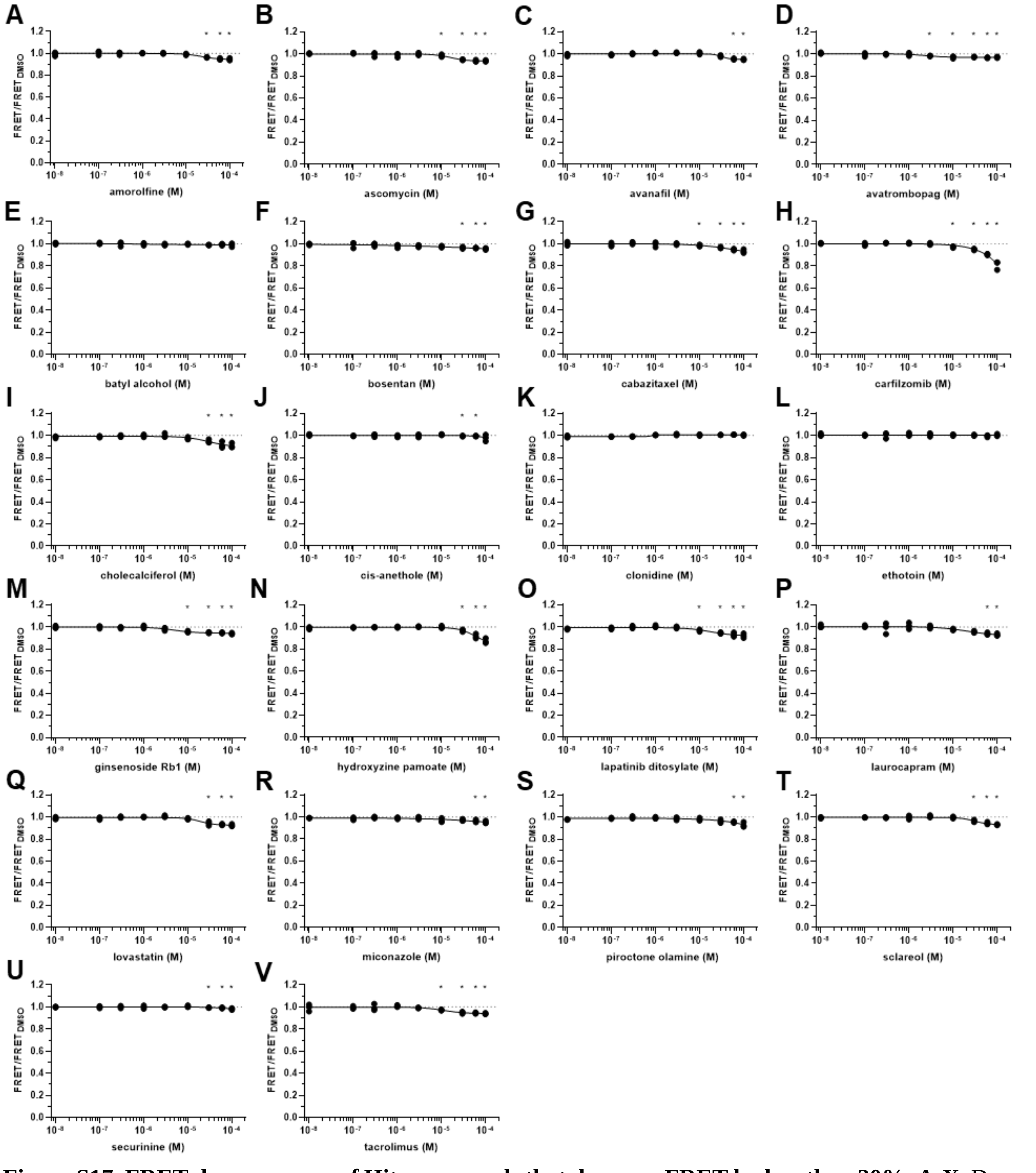

Figure S17. FRET dose response of Hit compounds that decrease FRET by less than 20%. A-X. Dose response of Hit compounds amorolfine (**A**), ascomycin (**B**), avanfil (**C**), avatrombopag (**D**), batyl alcholol (**E**), bosentan (**F**), cabazitaxel (**G**), carfilzomib (**H**), cholecalciferol (**I**), cis-anethole (**J**), clonidine (**K**), ethotoin (**L**), ginsenoside Rb1 (**M**), hydroxyzine pamoate (**N**), lapatinib ditosylate (**O**), laurocapram (**P**), lovastatin (**Q**), miconazole (**R**), piroctone olamine (**S**), sclareol (**T**), securinine (**U**) and tacrolimus (**V**) were tested on the in vitro ABD biosensor FRET. Data shown as individual data points, n=3.
